# Dynamic Control of a Multistate Chiral Supramolecular Polymer in Water

**DOI:** 10.1021/jacs.2c01063

**Published:** 2022-03-27

**Authors:** Fan Xu, Stefano Crespi, Gianni Pacella, Youxin Fu, Marc C. A. Stuart, Qi Zhang, Giuseppe Portale, Ben L. Feringa

**Affiliations:** † Stratingh Institute for Chemistry, University of Groningen, Nijenborgh 4, 9747 AG Groningen, The Netherlands; ‡ Zernike Institute for Advanced Materials, University of Groningen, Nijenborgh 4, 9747 AG Groningen, The Netherlands; § Key Laboratory for Advanced Materials and Joint International Research Laboratory of Precision Chemistry and Molecular Engineering, Feringa Nobel Prize Scientist Joint Research Center, Frontiers Science Center for Materiobiology and Dynamic Chemistry, Institute of Fine Chemicals, School of Chemistry and Molecular Engineering, East China University of Science and Technology, 200237 Shanghai, China

## Abstract

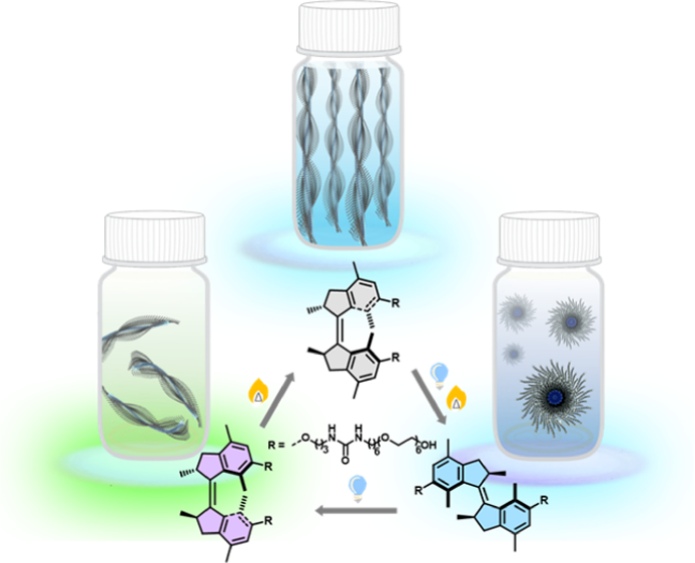

Natural systems transfer chiral information across multiple length scales through dynamic supramolecular interaction to accomplish various functions. Inspired by nature, many exquisite artificial supramolecular systems have been developed, in which controlling the supramolecular chirality holds the key to completing specific tasks. However, to achieve precise and non-invasive control and modulation of chirality in these systems remains challenging. As a non-invasive stimulus, light can be used to remotely control the chirality with high spatiotemporal precision. In contrast to common molecular switches, a synthetic molecular motor can act as a multistate chiroptical switch with unidirectional rotation, offering major potential to regulate more complex functions. Here, we present a light-driven molecular motor-based supramolecular polymer, in which the intrinsic chirality is transferred to the nanofibers, and the rotation of molecular motors governs the chirality and morphology of the supramolecular polymer. The resulting supramolecular polymer also exhibits light-controlled multistate aggregation-induced emission. These findings present a photochemically tunable multistate dynamic supramolecular system in water and pave the way for developing molecular motor-driven chiroptical materials.

## Introduction

Chirality is one of the most essential and fundamental features of nature.^1−8^ How chirality transfers from the molecular level to multiple length scales draws extensive attention and remains far from fully understood.^1−6,9−11^ Supramolecular interactions are crucial for building nanostructures and amplifying the asymmetry from the molecular level to the macroscopic scale.^12−17^ Controlling the supramolecular chirality in a self-assembled system requires a delicate match of many parameters, such as the distance between the chiral centers in the assembly and the interaction strength.^2−5,18^ As highly ordered assemblies are constructed through the use of a directional non-covalent bond, supramolecular polymers have a distinct advantage in transferring the chirality from monomers or environments (solvents and guests) to supramolecular handedness,^2^ playing an essential role in various areas^19−23^ such as amplification of asymmetry^16,24,25^ and optical and electronic devices.^26−28^

Controlling the chirality of supramolecular polymers using external stimuli, for example, heat,^29^ solvent,^30,31^ and light,^10,32−34^ holds enormous potential in smart and responsive materials. Among diverse stimuli, light exhibits superiority in controlling a system with high spatiotemporal precision,^35−38^ thus attracting increasing attention to the tuning of supramolecular chirality.^5,39^ However, most studies are based on two-stage photoswitches covalently linked to chiral moieties^10,32−34^ or coassembled with chiral molecules,^40,41^ and relatively few systems can be operated in water,^41^ a unique medium for assembly in nature. A multistate photoswitch with different inherent chiralities and distinct geometries would significantly enrich the opportunities of this field and allow more complex responsive functions in the future.^42−44^

Light-driven molecular motors undergo unidirectional rotation with interconvertible inherent chirality and a distinct helical geometry.^45−48^ Taking advantage of multistate chirality, they have been used to dynamically control metal–ligand helicate oligomers,^42^ cholesteric liquid crystal materials,^43^ spin selectivity,^49^ and asymmetric catalysis.^50−52^ However, the amplification of the asymmetry of molecular motors to the nanoscale and macroscopic scale still has massive untapped potential and has not been achieved in aqueous media so far.^42,43^

**Figure 1:**
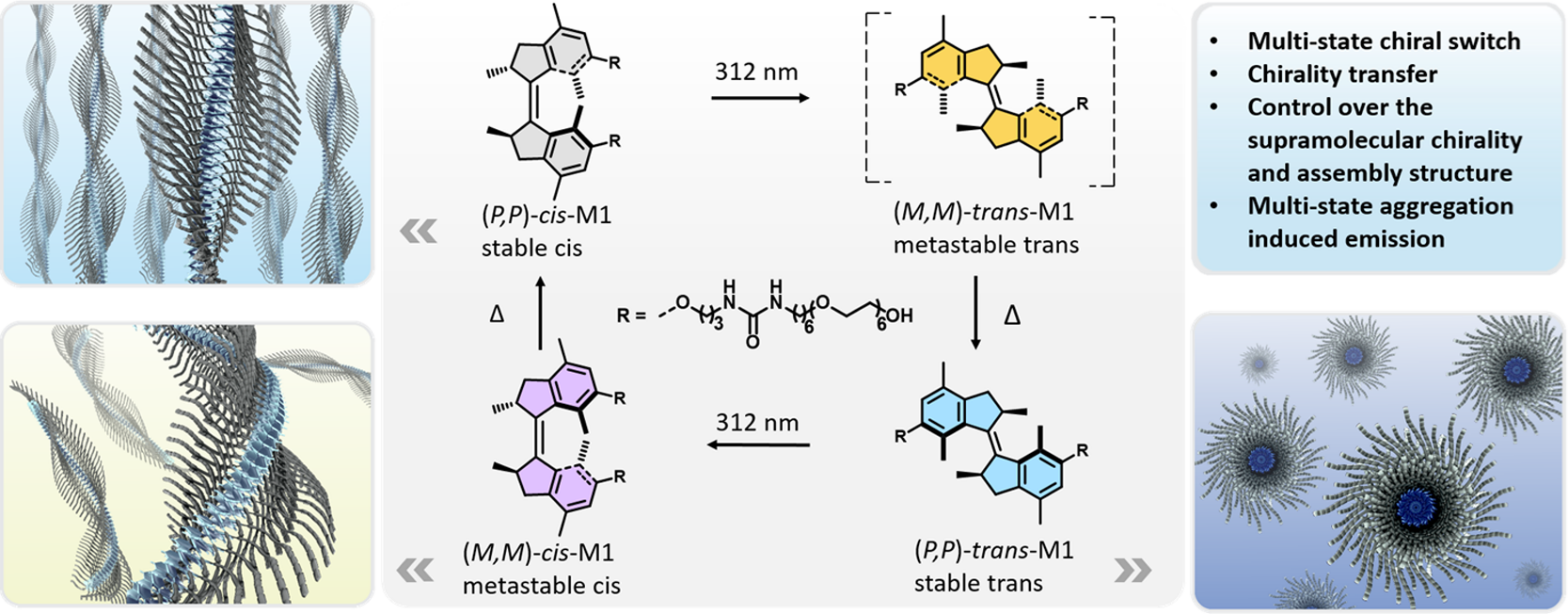
Molecular structures of molecular motor **M1** and illustration of multistate rotation, isomers with distinct chirality, and the corresponding assembly structures.

Here, we report water-soluble supramolecular polymers formed by molecular motors, including a racemic stable *cis*-motor (*cis*-**M1**) and the corresponding homochiral compound (*P*,*P*-*cis*-**M1**) (Figure 1). The chirality of the molecular motor was transmitted to the supramolecular polymer resulting in helical fibers in water. During the unidirectional rotation, the molecular motor displayed four states with different inherent chiralities and geometries, which allowed us to regulate the morphology and the chirality of the supramolecular polymer (Figure 1). Remarkably, the polymer exhibited light-tuneable multistate aggregation-induced emission (AIE), competing with the excited-state molecular motor rotation.

## Results and Discussion

### Molecular Design and Synthesis of Molecular Motors

Monomers were designed with an overcrowded alkene, first generation molecular motor^45−48^ core, and two urea groups bearing hexaethylene glycol and chains to ensure water solubility (Figure 1). The tight hydrogen bonding between urea groups is beneficial for constructing supramolecular polymers.^53−59^ C_6_ alkyl-linkers are positioned between the hexaethylene glycol chains and the urea groups, providing a hydrophobic pocket to facilitate hydrogen bonding. This bioinspired strategy of protecting hydrogen bonds in water has been shown by Meijer and others to favor the development of synthetic supramolecular polymers in aqueous media.^60−64^ The synthesis of racemic stable *cis*-**M1** and stable (*R*,*R*)-(*P*,*P*)-*cis*-**M1** is summarized in Supporting Information, Section S3 (Figure S1). The name of (*R*,*R*)-(*P*,*P*)-*cis*-**M1** is shortened as (*P*,*P*)-*cis*-**M1** in the present study. All the novel structures were characterized by ^1^H, ^13^C NMR, and high-resolution ESI-MS (Figures S25–S36).

The four-step rotary cycle process of **M1** features two photoisomerization and two thermal steps. Each photoisomerization is followed by a thermal helix inversion (THI): stable (*P*,*P*)-*cis*-**M1** photochemically converts to metastable (*M*,*M*)-*trans*-**M1**, which subsequently forms stable (*P*,*P*)-*trans*-**M1** during THI; then stable (*P*,*P*)-*trans*-**M1** photochemically converts to metastable (*M*,*M*)-*cis*-**M1**, followed by THI to recover stable (*P*,*P*)-*cis*-**M1** (Figure 1).

### Rotary Motion of Molecular Motors and Their Chirality Changes in Solution

Before studying the assembly and property of motors in water, we investigated the rotation of monomeric **M1** in MeOH using UV–vis, circular dichroism (CD) absorption, and ^1^H NMR spectroscopy. The monomeric state of **M1** in MeOH was confirmed by dynamic light scattering (DLS) measurements, showing an average hydrodynamic diameter (*D*_h_) around 2 nm (Figure S13). Upon 312 nm light irradiation at −15 °C, a characteristic absorption band of stable *cis*-**M1** at 270–330 nm decreased with the formation of an absorption band at 330–390 nm, showing an isosbestic point at 326 nm. This phenomenon indicated the selective photochemical interconversion of stable *cis*-**M1** to metastable *trans*-**M1** (Figure S2a). Keeping the molecule in the dark at −15 °C, the absorption band at 330–390 nm diminished with an increase in absorption at 260–330 nm, as a consequence of the THI interconversion of metastable *trans* to stable *trans* (Figure S2b).

Subsequent irradiation with 312 nm light led to a decrease of the absorption band at 260–330 nm and an increase of the one at 330–400 nm, indicating the selective photoisomerization of stable *trans*-**M1** to metastable *cis*-**M1** (Figure S2c). After warming the sample at 45 °C in the dark, the absorption band at 330–400 nm diminished with an increase at 270–330 nm, suggesting the helix inversion of metastable *cis*-**M1** to stable *cis*-**M1** (Figure S2d). The rotation behavior of **M1** is comparable with the one observed in a previous study in our group on first generation molecular motor-based double-stranded helicates.^42^ Eyring analysis of the THI data of metastable *trans*-**M1** to stable *trans*-**M1** and metastable *cis*-**M1** to stable *cis*-**M1** revealed a standard Gibbs free energy of activation (Δ^⧧^*G*°) of 78.8 and 100.4 kJ mol^–1^ at 20 °C, respectively (Figures S3 and S4). The determined half-life of metastable *cis*-**M1** is 24.8 h, while the one of metastable *trans*-**M1** is 12.4 s, indicating the challenge to capture metastable *trans*-**M1** in solution at room temperature.

**Figure 2:**
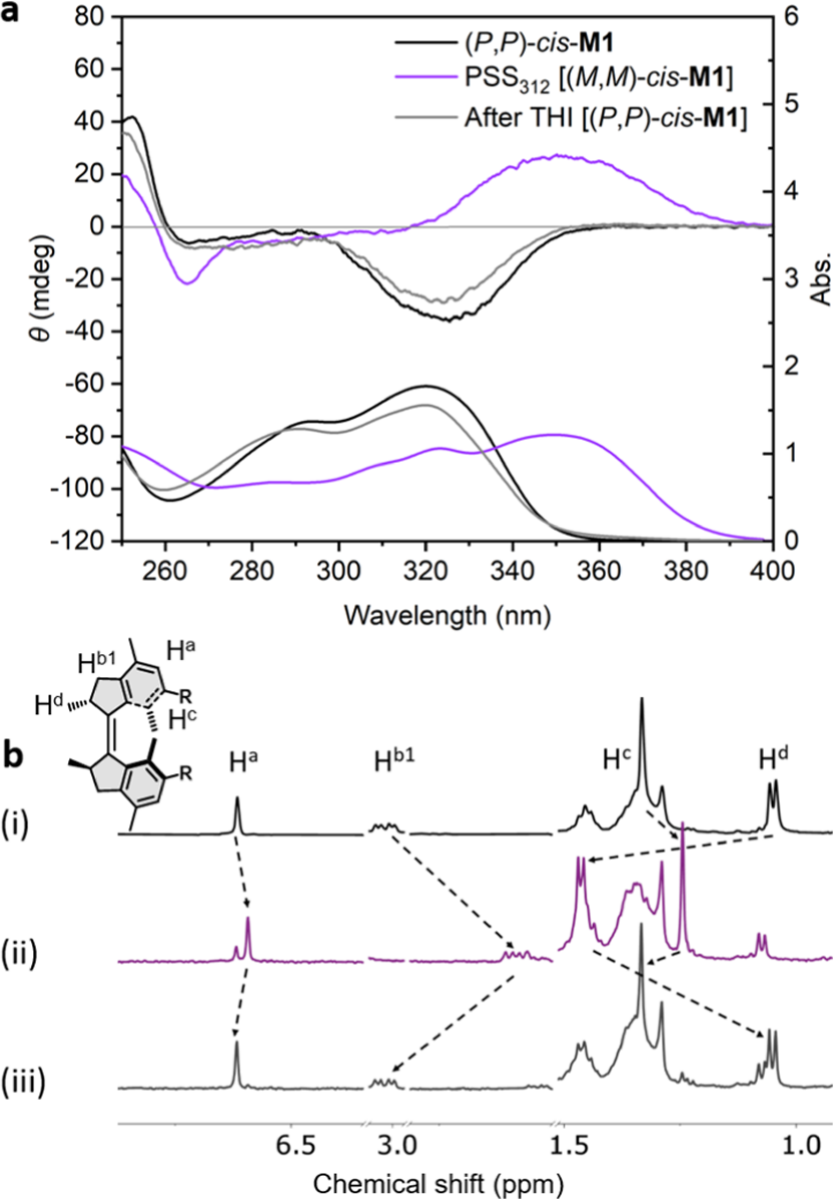
(a) Changes in the UV–vis (right axis) and CD (left axis) absorption spectra (MeOH, 293 K) of (*P*,*P*)-*cis*-**M1** (78 μM) upon irradiation with 312 nm light for 30 min to reach the PSS and subsequently removing the light source and warming at 45 °C for 5 h to allow THI; (b) changes in the ^1^H NMR spectra of (i) (*P*,*P*)-*cis*-**M1** (2 mM), (ii) upon irradiation with 312 nm light for 30 min at 293 K to the PSS, and (iii) subsequently removing the light source and warming at 55 °C for 1.5 h to induce THI.

Due to the short half-life of metastable *trans*-**M1**, irradiation of stable (*P*,*P*)-*cis*-**M1** at 20 °C resulted in the generation of metastable *cis*-**M1** at the photostationary state (PSS), indicated by the decrease of the absorption band at 270–330 nm, accompanied by the increase of the absorption band at 330–400 nm (Figure 2a). Stable (*P*,*P*)-*cis*-**M1** displayed a characteristic negative signal at 290–350 nm in the CD spectrum, which disappeared with the generation of a positive band at 320–400 nm upon irradiation, characteristic of the opposite inherent chirality of (*M*,*M*)-*cis*-**M1** and (*P*,*P*)-*cis*-**M1**. Nearly identical CD and absorption spectra of (*P*,*P*)-*cis*-**M1** were recovered after keeping the sample in the dark at 45 °C for 5 h, indicating a THI process of metastable (*M*,*M*)-*cis* to stable (*P*,*P*)-*cis*-**M1**. The same isomerization process was also demonstrated by ^1^H NMR (Figure 2b). Proton signals of H^d^ (δ = 1.05 ppm) shift downfield to 1.46 ppm, while H^a^, H^b^, and H^c^ shift upfield upon 312 nm light irradiation for 30 min [Figure 2b(i,ii)], in accordance with the conversion from stable *cis*-**M1** to metastable *cis*-**M1**. The ratio of metastable *cis* to stable *trans*-**M1** is 76:24 at the PSS, established by integrating the NMR signals. Subsequently, warming the sample at 55 °C in the dark for 1.5 h resulted in the recovery of the initial ^1^H NMR spectrum of stable *cis*-**M1** [Figure 2b(iii)].

### Assembly in Water and Chirality Transfer from Molecular Motors to Supramolecular Polymers

**Figure 3:**
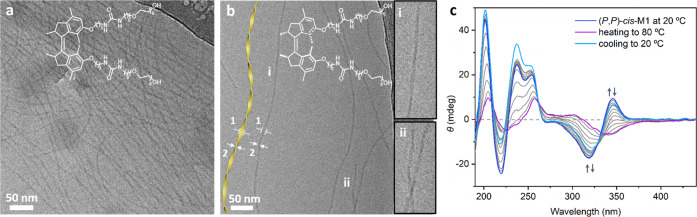
Cryo-TEM images of the assembly structure in water of (a) racemic stable *cis*-**M1** (3 mg/mL) and (b) (*P*,*P*)-*cis*-**M1** (1 mg/mL), inset: enlarged images at positions i and ii (additional images are shown in the Supporting Information). (c) Temperature-dependent CD spectra of (*P*,*P*)-*cis*-**M1** (52 μM) in water.

To test if the molecular motor can result in ordered assembly structures, an aqueous solution of racemic stable *cis*-**M1** was first characterized by cryogenic electron transmission microscopy (cryo-TEM). To our delight, racemic stable *cis*-**M1** was found to form fibers with a uniform diameter of 7.0 ± 0.8 nm and over micrometers in length (Figure 3a). As stable *cis*-**M1** formed a well-organized 1D assembly, we envisioned that the enantiomerically pure stable *cis*-**M1** holds promise to transfer its molecular chirality to a supramolecular polymer. Consequently, we conducted the subsequent assembly study starting from (*P*,*P*)-*cis*-**M1**.

Indeed, the cryo-TEM images revealed that (*P*,*P*)-*cis*-**M1** formed uniform fibers with micrometer length (Figures 3b and S14). These fibers showed a regular variation in the width along the axis under cryo-TEM, indicating the helical structure of the supramolecular polymer. The 1/2 pitch was around 70 nm. The maximum width is 7.8 ± 0.7 nm (Figure 3b, label 1) and the minimum is 3.8 ± 0.4 nm (Figure 3b, label 2). The identical sample was characterized in water by small angle X-ray scattering (SAXS). The SAXS profile shows a *q*^–1^ slope of the curve at low *q*-values in the log–log plot, suggesting a rod (fiber)-like structure (vide infra, Figure 6, black circle). The curve was fitted with the analytical model for flexible cylindrical objects, resulting in an average diameter of 5.2 ± 0.4 nm. The total length and the Kuhn length of the structures are beyond the resolution of the measurement (>60 nm). Despite the fact that this diameter refers to a solid rod-like object, thus showing a slight deviation from the shape of twisted fiber, it is in agreement with the cryo-TEM observations. We further characterized the supramolecular polymer by Fourier transform infrared (FTIR) spectroscopy. The strong vibrational band centered at 3337 cm^–1^ belongs to hydrogen-bonded N–H in the urea moieties (Figure S9, black curve), confirming that the hydrogen bonds contributed to forming a supramolecular polymer of (*P*,*P*)-*cis*-**M1**.

The presence of helical fibers encouraged us to study the chirality transfer process by temperature-dependent CD spectra. Differently from the CD spectrum in MeOH, with a characteristic negative signal at 290–350 nm, an aqueous solution of (*P*,*P*)-*cis*-**M1** showed a positive Cotton effect at 280–360 nm at 20 °C, suggesting chiral helical packing of molecules (Figure 3c). No linear dichroism (LD) signal was measured for the sample, indicating that the CD signals are induced from molecules to supramolecular polymers (Figure S10).^65^ Upon heating, the positive Cotton effect diminished with an appearance of a negative band similar to that in MeOH, implying a disorder of the supramolecular polymer and the disappearance of the supramolecular chirality (Figures 3c and S11). After cooling and stabilizing at 20 °C for 2 h, the positive Cotton effect was recovered, suggesting the reformation of the chiral supramolecular polymer (Figure 3c). These features confirmed the chirality transfer from the molecular motor (*P*,*P*)-*cis*-**M1** to the supramolecular polymer. Temperature-dependent DLS measurements revealed the changes in the size of the assemblies upon heating and cooling, confirming the disorder and reformation of the supramolecular polymers (Figure S12). In addition, a LCST-type phase separation was found in the heating process with the critical temperature at ca. 60 °C (Supporting Information, Section S9).

### Rotation of Molecular Motors in the Supramolecular Polymer in Water

**Figure 4:**
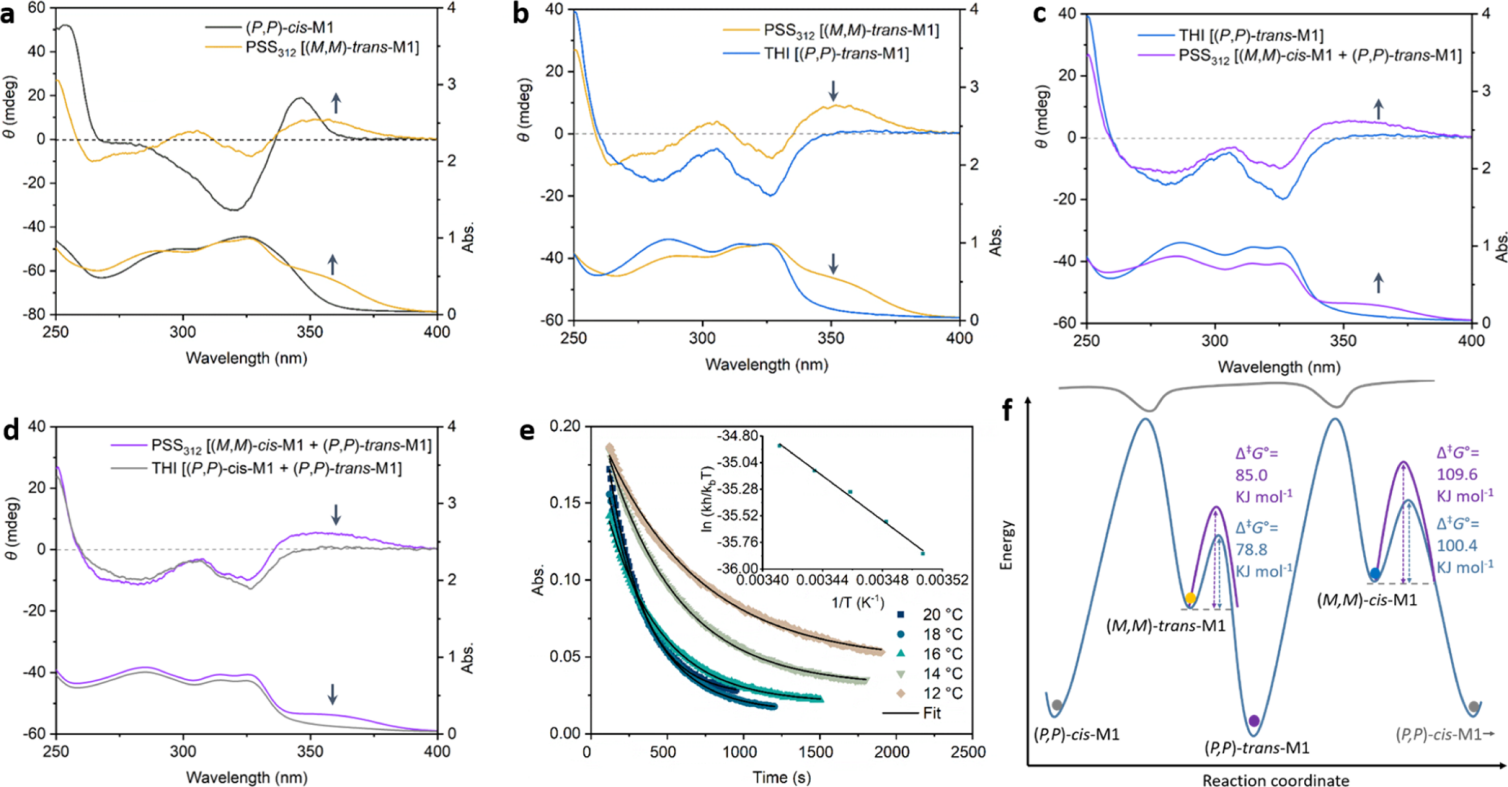
CD and UV–vis spectra of (a) (*P*,*P*)-*cis*-**M1** (78 μM) after irradiation with 312 nm light for 2 min to get the PSS, (*M*,*M*)-*trans*-**M1**, (b) after removing the light source and keeping 20 °C for 10 min to get (*P*,*P*)-*trans*-**M1**, (c) after subsequent irradiation with 312 nm light for 10 min to get a PSS mixture of (*P*,*P*)-*trans*-**M1** and (*M*,*M*)-*cis*-**M1** in water, (d) after removing the light source and warming at 45 °C for 3 days, processing the THI of (*M*,*M*)-*cis*-**M1** to get (*P*,*P*)-*cis*-**M1** (293 K, 1 cm cuvette), and (e) time-dependent absorption changes at 365 nm during the THI of metastable (*M*,*M*)-*trans*-**M1** in water at different temperatures, inset: Eyring plots with a liner fit. (f) Energy diagram of the rotation of molecular motor in MeOH and water, respectively.

The motion of molecular motors in confined space is crucial for their applications in materials.^66−68^ To study the rotation of the molecular motors inside the aqueous supramolecular polymer, we monitored the rotation process by CD and UV–vis absorption spectroscopy. Interestingly, we observed the appearance of the metastable *trans* isomer (*M*,*M*)-*trans*-**M1** at 20 °C (Figure 4a,b). The *trans* isomer (*M*,*M*)-*trans*-**M1** was not observed in MeOH at the same temperature, in which the motors are molecularly dissolved. After exposing (*P*,*P*)-*cis*-**M1** to 312 nm light for 2 min, the CD spectrum displayed a positive signal at 330–390 nm, and the UV–vis absorption spectrum showed a band at 330–390 nm, suggesting the presence of metastable (*M*,*M*)-*trans*-**M1** (Figure 4a). After keeping in the dark at 20 °C for 10 min, the absorption band and positive CD signal at 330–390 nm disappeared with an increase of the absorption band and negative CD signal at 270–330 nm, indicating that the THI of metastable (*M*,*M*)-*trans*-**M1** to stable (*P*,*P*)-*trans*-**M1** was completed (Figure 4b). Eyring analysis of the THI in water (aggregate states) revealed a Δ^⧧^*G*° of 85.0 kJ mol^–1^, which was higher than 78.8 kJ mol^–1^ in the monomeric state (Figure 4e,f). The increased energy barrier results in a longer half-life of 2.6 min, benefitting from the fact that metastable (*M*,*M*)-*trans*-**M1** is stabilized in the fibers.

Much to our delight, we obtained the pure stable *trans* isomers after irradiation and THI in water, as revealed by integrating the characteristic signals in ^1^H NMR (Figure S7b). Hence, the original (*P*,*P*)-*cis*-**M1** has been fully converted to (*P*,*P*)-*trans*-**M1**. Upon subsequent irradiation with 312 nm light for 10 min, the absorption band and negative CD signal at 270–330 nm decreased with the generation of an absorption band and positive CD signal at 330–400 nm, suggesting the isomerization of stable (*P*,*P*)-*trans*-**M1** to metastable (*M*,*M*)-*cis*-**M1** (Figure 4c). The ^1^H NMR spectra of the identical sample revealed a ratio of (*M*,*M*)-*cis*-**M1** and (*P*,*P*)-*trans*-**M1** as 32:68 (Figure S7c), which is lower than the one in MeOH (Figure 2b). After warming at 45 °C for 3 days, the absorption band and positive CD signal at 330–400 nm disappeared, suggesting the THI of (*M*,*M*)-*cis*-**M1** to (*P*,*P*)-*cis*-**M1** (Figure 4d). The complete conversion of (*M*,*M*)-*cis*-**M1** by THI to the original isomer was confirmed by ^1^H NMR (Figure S7d), showing the four-stage rotary cycle. Eyring analysis of the THI revealed a Δ^⧧^*G*° of 109.6 kJ mol^–1^ and a *t*_1/2_ of 44.5 days at 20 °C or 19.4 h at 45 °C, which were higher than those found in MeOH (Figures 4f and S5).

### Multistate Chirality and Morphology Changes of Supramolecular Polymers

**Figure 5:**
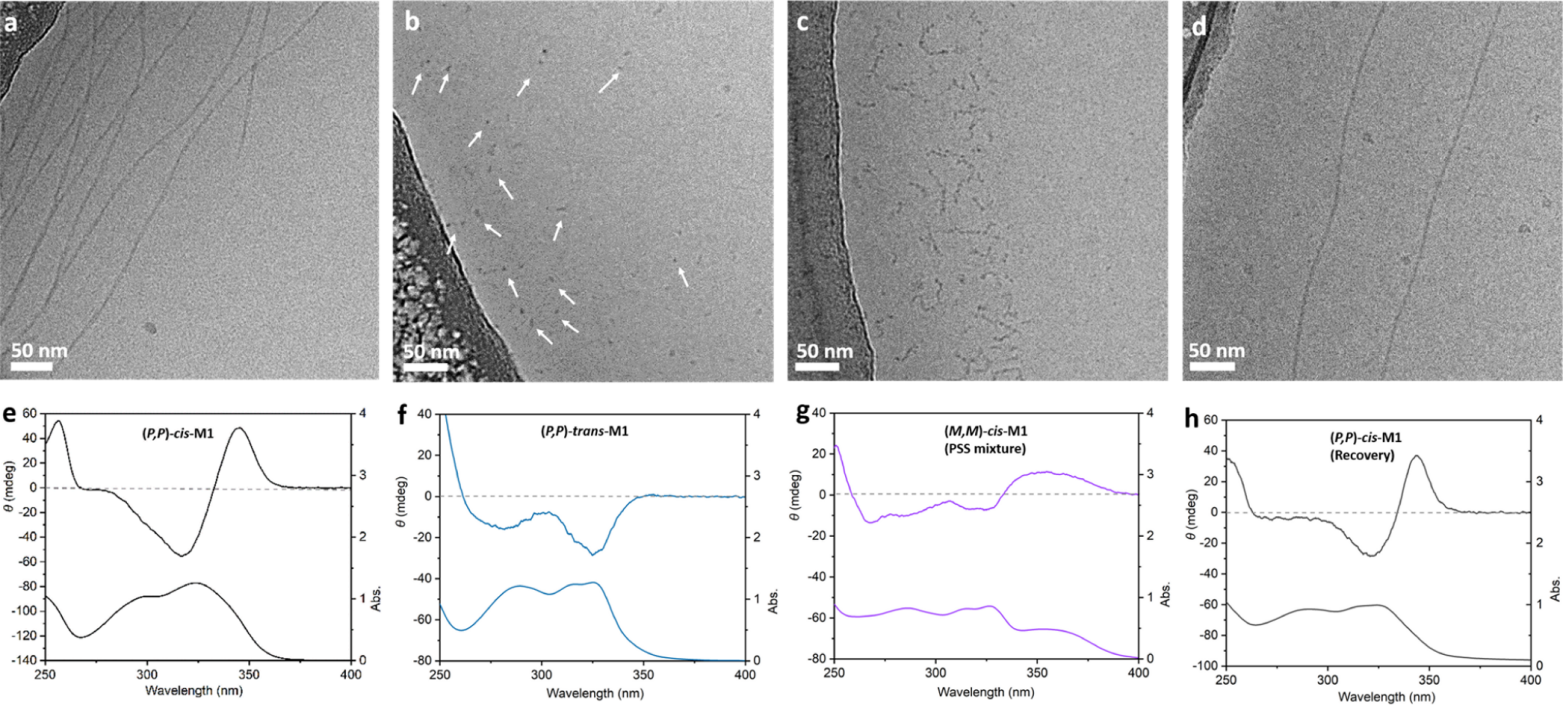
Cryo-TEM images of (a) (*P*,*P*)-*cis*-**M1** (780 μM), (b) after irradiation with 312 nm light for 2 min and keeping in the dark for 10 min to reach (*P*,*P*)-*trans*-**M1** (micelles were pointed out with arrows for clearance, and not all the micelles were pointed), (c) after subsequent irradiation with 312 nm light for 10 min to get a PSS mixture of (*M*,*M*)-*cis*-**M1** and (*P*,*P*)-*trans*-**M1** in water, and (d) (*P*,*P*)-*trans*-**M1** after irradiation with 312 nm light for 10 min and keeping in the dark at 45 °C for 5 h in a water/THF (7/3) mixture to recover (*P*,*P*)-*cis*-**M1**, followed by repreparation in pure water. (e–h) CD and UV–vis absorption spectra of the identical samples in (a–d), respectively (293 K, 1 mm cuvette).

**Figure 6:**
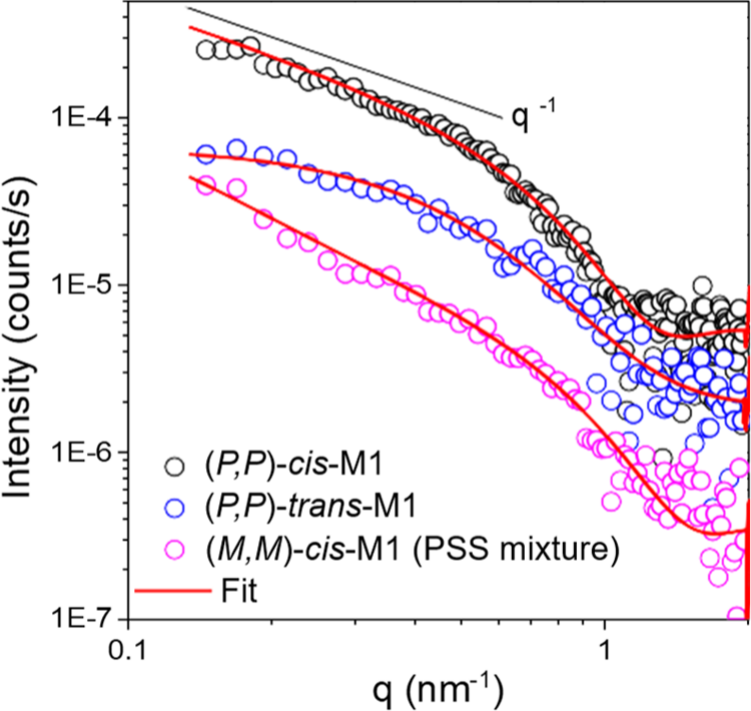
SAXS profiles for the supramolecular polymer of (*P*,*P*)-*cis*-**M1** in water before (black circle) and after irradiation with 312 nm light for 2 min and keeping in the dark for 10 min to reach (*P*,*P*)-*trans*-**M1** (blue circle), subsequent irradiation with 312 nm light for 10 min to get a PSS mixture of (*M*,*M*)-*cis*-M1 and (*P*,*P*)-*trans*-**M1** (purple circle). The red curves are the fittings with the Guinier approximation (Supporting Information Section S11).

To explore the supramolecular chirality and morphology changes of the assemblies during the rotation of the molecular motor, we performed cryo-TEM, SAXS, CD, and UV–vis absorption measurements at different stages. As presented above, (*P*,*P*)-*cis*-**M1** formed helical fibers and showed a positive Cotton effect at 280–360 nm in the CD spectra (Figures 5a,e and S14). The fibers of (*P*,*P*)-*cis*-**M1** transformed into micelles after irradiation for 2 min and subsequently keeping in the dark for 10 min to reach (*P*,*P*)-*trans*-**M1** (Figures 5b,f and S15). The SAXS profile of (*P*,*P*)-*trans*-**M1** exhibits a significant decrease in intensity at low *q*-values, and the *q*^–1^ slope of the (*P*,*P*)-*cis*-**M1** sample is not observed anymore, suggesting the presence of less-elongated assemblies (Figure 6, blue circle). The average diameter of the aggregates as determined using the fit of the SAXS profile is 5.0 ± 0.4 nm, which is in good agreement with the cryo-TEM study result (5.7 ± 0.7 nm). After irradiation for 10 min, cryo-TEM images showed worm-like fibers with a diameter of 6.2 ± 0.8 nm, which were formed from the mixture of (*M*,*M*)-*cis* and (*P*,*P*)-*trans*-**M1** (Figures 5c,g and S16). A SAXS pattern of the same sample displays an increase of the intensity at low *q*-values again, indicating the existence of more extended assemblies than spherical micelles formed by (*P*,*P*)-*trans*-**M1** (Figure 6 purple circle). Data analysis revealed a diameter of 4.8 ± 0.4 nm and a Kuhn length (*L*_Kuhn_) around 20 nm, comparable to the observations from the cryo-TEM images. As described above, the ratio of (*M*,*M*)-*cis* and (*P*,*P*)-*trans*-**M1** is 32:68 at the PSS in water. A considerable amount of (*P*,*P*)-*trans*-**M1** remaining unconverted after THI might affect the recovery of the helical fibers. Indeed, the morphology of aggregates after THI of (*M*,*M*)-*cis* to (*P*,*P*)-*cis*-**M1** remained as worm-like fibers, although almost all the (*M*,*M*)-*cis*-**M1** have converted to (*P*,*P*)-*cis*-**M1** (Figures S17 and S18). To improve the ratio of (*M*,*M*)-*cis*-**M1** and (*P*,*P*)-*trans*-**M1** at the PSS and speed up the following THI, we performed the irradiation and warming in a water/THF (7/3) mixture. In this way, the ratio of (*M*,*M*)-*cis*-**M1** and (*P*,*P*)-*trans*-**M1** at the PSS increases to 70:30, revealed by integrating ^1^H NMR signals (Figure S8a). Helical fibers were recovered by preparing the above sample in water, as evidenced from the cryo-TEM images and the positive Cotton effect in the CD spectrum (Figures 5d,h and S19).

### Light-Controllable Multistate AIE

**Figure 7:**
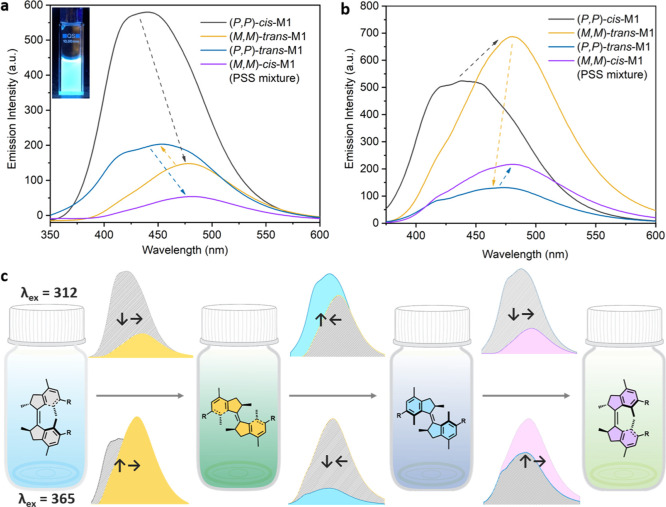
Changes in the fluorescence spectra of (*P*,*P*)-*cis*-**M1** (78 μM) during multistate rotation in water upon the excitation at (a) λ_ex_ = 312 nm, inset: an image of (*P*,*P*)-*cis*-**M1** upon the irradiation at λ_ex_ = 312 nm in water, and (b) λ_ex_ = 365 nm. (c) Schematic illustration of light-tuneable multistate AIE upon excitation at λ_ex_ = 312 nm and λ_ex_ = 365 nm, respectively.

The supramolecular polymer of (*P*,*P*)-*cis*-**M1** showed a blue emission centered at 440 nm upon excitation at λ_ex_ = 312 nm in water (Figure 7a). (*P*,*P*)-*cis*-**M1** show no emission in the monomeric state, as suggested by the absence of fluorescence in MeOH (Figure S20). To identify the lowest concentration for AIE, we plotted the concentration-dependent fluorescence intensity. This value increased with the concentration after a sharp transition at a critical concentration of 6 μM (Figure S21). As the mechanism of AIE is associated with the restriction of the intramolecular rotation,^69^ we assumed that the emission of our supramolecular polymer was attributed to the restriction of the excited-state rotation of the molecular motor in confined space. Gratifyingly, this increased barrier is not enough to halt the photochemical isomerization of the molecular motors inside the supramolecular assembly.

These results encouraged us to study the multistate photoresponsive AIE. We measured the fluorescence spectra of (*P*,*P*)-*cis*-**M1** (78 μM) during the four-stage rotation under the excitation of 312 nm light (Figure 7a). After irradiation with 312 nm for 2 min, the emission centered at 440 nm decreased and red-shifted to 480 nm due to the presence of metastable (*M*,*M*)-*trans*-**M1**. The emission shifted in the blue region and increased at 440 nm after keeping the sample in the dark at room temperature for 10 min to reach stable (*P*,*P*)-*trans*-**M1**. After subsequent irradiation of (*P*,*P*)-*trans*-**M1**for 10 min to the PSS, the emission red-shifted to 480 nm again, which was attributed to a mixture of metastable (*M*,*M*)-*cis*-**M1** and stable (*P*,*P*)-*trans*-**M1**. The differences in emission intensity might be attributed to the packing of molecules in the aggregates, while the emission wavelength is related to the Stokes shift of different absorption bands.^70−72^

Notably, the four states of **M1** also showed the emission upon excitation with 365 nm light (Figure 7b). The emission wavelengths of the four states were comparable to those observed when exciting at 312 nm, while the emission intensities showed an inverse trend (Figure 7c). An increase of the emission intensity was observed in the transition of (*P*,*P*)-*cis* to (*M*,*M*)-*trans*-**M1** and (*P*,*P*)-*trans* to (*M*,*M*)-*cis*-**M1**, and a decrease of the emission intensity was found for the THI of (*M*,*M*)-*trans* to (*P*,*P*)-*trans*-**M1**. In addition, (*P*,*P*)-*cis* and (*P*,*P*)-*trans*-**M1** show a relatively lower emission intensity, while (*M*,*M*)-*trans* and (*M*,*M*)-*cis*-**M1** show a stronger emission intensity under λ_ex_ = 365 nm compared to those upon 312 nm excitation. To avoid the possible photoinduced rotation of **M1** during the measurements, the fluorescence quantum yield (Φ_em_) was measured upon the excitation of their unfavorable excitation wavelength (Supporting Information Section S14, Table S1).^73−75^ (*P*,*P*)-*cis* and (*P*,*P*)-*trans*-**M1** were measured under λ_ex_ = 365 nm; (*M*,*M*)-*trans* and (*M*,*M*)-*cis*-**M1** were characterized under λ_ex_ = 312 nm. Φ_em_ of (*P*,*P*)-*cis* reaches 9.2%, even under an unfavorable excitation wavelength. The other three states show relatively lower Φ_em_, 3.2, 3.4, and 1.4%, under their unfavorable excitation wavelengths, respectively (Table S1). Emission lifetimes of the aggregates formed by (*P*,*P*)-*cis*-**M1**, (*P*,*P*)-*trans*-**M1**, and (*M*,*M*)-*cis*-**M1** are shorter than 0.5 ns (Supporting Information Section S15, Figures S22–S24). Distinct from the major current two-state strategy,^76^ our work presented a unique and facile way toward multistate light-controllable AIE materials.

## Conclusions

In conclusion, we developed a multistate photoresponsive supramolecular polymer in water based on synthetic molecular motor **M1**. The chirality of the molecular motor (*P*,*P*)-*cis*-**M1** successfully transferred to the supramolecular polymer in an aqueous solution. The rotation-induced intrinsic chirality and geometric changes dramatically influenced the morphology and chirality of the supramolecular polymer, thus achieving dynamic control of the supramolecular polymer in multiple self-assembled states. Starting from the helical fibers, the morphology was transformed into micelles, followed by worm-like micelles, and finally the helical fibers were recovered. The resulting assemblies showed unique multistate AIE, which could be tuned by light. The present study showed a multistate supramolecular system in water with light-controllable properties and established the basis for developing advanced multifunctional and responsive molecular motor-based chiroptical materials.

## Supporting Information Available

Synthesis; experimental conditions for UV–vis, CD, cryo-TEM, FTIR, and SAXS; fluorescence quantum yield and emission lifetime measurements; Eyring analysis; computational study; and ^1^H, ^13^C NMR, and HRMS data (PDF)
