# Supplementary material for: Dynamic Control of a Multistate Chiral Supramolecular Polymer in Water
Source: J Am Chem Soc. 2022 Mar 27;144(13):6019–27. doi: 10.1021/jacs.2c01063 (PMC8991000; doi:10.1021/jacs.2c01063)
Supplement: Supplementary file 1 — ja2c01063_si_001.pdf [file ja2c01063_si_001.pdf]

# Supporting Information

# Dynamic control of a multistate chiral supramolecular polymer in water

Fan Xu,[†] Stefano Crespi,[†] Gianni Pacella,[‡] Youxin Fu,[†] Marc C. A. Stuart,[†] Qi Zhang,[†] Giuseppe Portale,[‡] Ben L. Feringa*[,†,‡,§]

[†]Stratingh Institute for Chemistry, University of Groningen, Nijenborgh 4, 9747 AG Groningen, The Netherlands.

[‡]Zernike Institute for Advanced Materials, University of Groningen, Nijenborgh 4, 9747 AG Groningen, The Netherlands.

[§]Key Laboratory for Advanced Materials and Joint International Research Laboratory of Precision Chemistry and Molecular Engineering, Feringa Nobel Prize Scientist Joint Research Center, Frontiers Science Center for Materiobiology and Dynamic Chemistry, Institute of Fine Chemicals, School of Chemistry and Molecular Engineering, East China University of Science and Technology, Shanghai 200237, China.

*To whom correspondence should be addressed.

E-mail: b.l.feringa@rug.nl (B.L.F.)

**Table of Contents**

1. Materials. ........ 3
2. General. ........ 3
3. Synthesis. ........ 4
4. UV-vis spectroscopic study on the rotation of molecular motors. ........ 6
5. Eyring analysis on the thermal helix inversion of molecular motors. ........ 7
6. $^1$H NMR study on the rotation of molecular motors. ........ 8
7. Fourier transform infrared spectroscopy (FTIR). ........ 10
8. Linear dichroism (LD) study. ........ 10
9. Temperature-dependent dynamic light scattering (DLS) measurements ........ 10
10. Cryo-TEM study. ........ 12
11. SAXS measurements. ........ 18
12. Fluorescence spectra. ........ 19
13. Concentration-dependent emission. ........ 19
14. Fluorescence quantum yield. ........ 19
15. Emission lifetime measurements ........ 20
16. NMR and HRMS data. ........ 22
References: ........ 28

## 1. Materials.

All commercially available chemicals were purchased from Acros, Aldrich, or TCI, and were used as received. Solvents used in the reactions were dried using an MBraun SPS-800 solvent purification system or purchased from Acros. The water (ULC/MS grade) used in sample preparation was purchased from Biosolve. Solvents used in the irradiation experiments were degassed by purging with argon for 30 min. Analytical TLC was carried out on Merck silica gel 60 F254 plates, and visualization was accomplished under UV light.

## 2. General.

Column chromatography was performed on a Reveleris X2 Flash Chromatography system. NMR spectra were recorded at 25 °C on Varian AMX400 ($^1$H: 400 MHz, $^{13}$C: 101 MHz), Varian Unity Plus ($^1$H: 500 MHz, $^{13}$C: 125 MHz) and Bruker Avance ($^1$H: 600 MHz, $^{13}$C: 151 MHz) spectrometers. Chemical shifts ($\delta$) are expressed relative to the resonances of the residual non-deuterated solvent for $^1$H NMR [$CDCl_3$: $^1$H(δ) = 7.26 ppm] and $^{13}$C NMR [$CDCl_3$: $^{13}$C(δ) = 77.2 ppm]. Absolute values of the coupling constants are given in Hertz (Hz), regardless of their sign. Multiplicities are abbreviated as singlet (s), doublet (d), doublet of doublets (dd), triplet (t), triplet of doublets (td), quartet (q), multiplet (m), and broad (br). High-resolution mass spectrometry (HRMS) was performed on an LTQ Orbitrap XL spectrometer with ESI ionization. All reactions were performed under anhydrous conditions under an $N_2$ atmosphere. UV-vis spectra were recorded on a Hewlett-Packard HP 8543 spectrometer in a quartz cuvette with 1 cm path length. CD spectra were recorded on Jasco J-815 Circular Dichroism Spectrometer. The emission spectra were recorded from 350–600 nm by using a JASCO FP6200 spectrofluorometer. The fluorescence quantum yield was measured by an Edinburgh instrument FS5 spectrofluorometer. Irradiation of samples was carried out at 298 K using a Spectroline hand-held UV lamp with LONGLIFE™ filter (8-watt model, 312 nm) positioned at a distance of 2 cm from the sample. An FEI T20 cryo-electron microscope equipped with a Gatan model 626 cryo-stage was used to record the morphology of supramolecular polymers, operating at 200 kV under low-dose conditions with a slow-scan CCD camera. FTIR patterns were measured on a PerkinElmer Spectrum 400. SAXS measurements were performed using the Multipurpose Instrument for Nanostructure Analysis (MINA) diffractometer at the University of Groningen equipped with Cu rotating anode. DLS measurements are conducted on Zetasizer Ultra equipment with a fluorescence filter using He-Ne laser (633 nm). The aqueous sample solution was placed in a plastic cuvette, while the sample in MeOH was placed in a glass cuvette. All the setups were calibrated before measurements. For each temperature, five measurements were performed after equilibrium for 300 s. The highest temperature is 70 °C for the equipment and settings. Data were analyzed in the ZS XPLORER software, assuming a refractivity index of 1.56. The size of assemblies was determined following the number distribution. Optical rotation was measured on a SCHMIDT + HAENSCH Polartronic MH8 polarimeter at 589 nm (sodium D line), 20 °C, reported as: $[\alpha]_D^T$ (concentration (g/100 mL), in corresponding solvent). Optical rotation of (*P*,*P*)-*cis*-**M1**: $[\alpha]_D^{20}$= – 65.7° (c = 1.02, $CH_2Cl_2$).

**3. Synthesis.**

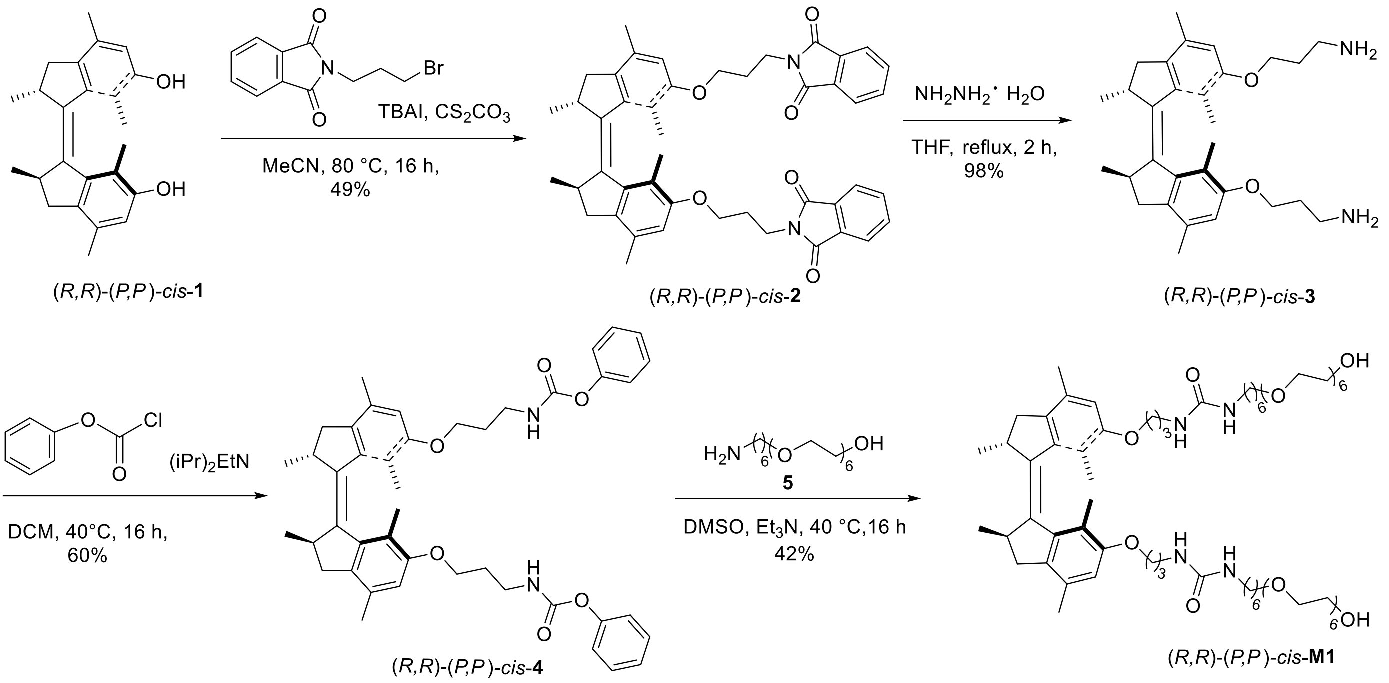


**Figure S1**. Synthesis scheme of (*P,P*)-*cis*-**M1**.

(*P,P*)-*cis*-**2**

To a suspension of (*P,P*)-*cis*-**1**[1] (600 mg, 1.72 mmol) in MeCN (10 mL) was added 2-(3-bromopropyl) isoindoline-1,3-dione (1.38 g, 5.16 mmol), tetrabutylammonium iodide (1.91g, 5.16 mmol) and $Cs_2CO_3$ (1.68 g, 5.16 mmol). The mixture was stirred at 80 °C overnight followed by concentrating *in vacuo*. To the concentrated mixture was added 20 mL deionized water. The aqueous phase was extracted with EtOAc. The organic layer was washed with brine, dried over $Na_2SO_4$ and concentrated *in vacuo*. The crude product was purified by column chromatography ($SiO_2$, EtOAc:pentane = 1:4) to afford compound (*P,P*)-*cis*-**2** (610 mg, 0.84 mmol, 49%) as a white solid. $^1$H NMR (400 MHz, $CDCl_3$) δ 7.77 (dd, *J* = 5.4, 3.1 Hz, 4H), 7.65 (dd, *J* = 5.4, 3.0 Hz, 4H), 6.52 (s, 2H), 4.05 (dt, *J* = 9.5, 6.1 Hz, 2H), 3.92 (dt, *J* = 9.5, 6.2 Hz, 2H), 3.86 (t, *J* = 7.2 Hz, 4H), 3.32 (p, *J* = 6.6 Hz, 2H), 3.03 (dd, *J* = 14.5, 6.3 Hz, 2H), 2.36 (d, *J* = 14.5 Hz, 2H), 2.24 (s, 6H), 2.17 – 2.09 (m, 4H), 1.41 (s, 6H), 1.06 (d, *J* = 6.7 Hz, 6H).$^{13}$C NMR (101 MHz, $CDCl_3$) δ 168.4, 155.7, 142.3, 141.1, 136.4, 133.9, 132.3, 130.5, 123.3, 122.7, 111.8, 66.1, 42.0, 38.3, 35.6, 29.0, 20.7, 19.0, 14.4. HRMS (ESI+) calcd. for $[M+H]^+$: 724.3457, found: 724.3462.

(*P,P*)-*cis*-**3**

To a suspension of (*P,P*)-*cis*-**2** (560 mg, 0.77 mmol) in EtOH (20 mL) was added hydrazine hydrate (50–60%, 15.4 mmol, 0.9 mL), following by heating the mixture at reflux for 2 h. After cooling and concentrating *in vacuo*, the mixture was dissolved in 15% aq. NaOH (20 mL), and extracted with DCM. The organic layer was washed with brine and dried over $Na_2SO_4$, and then concentrated *in vacuo* to afford (*P,P*)-*cis*-**3** (350 mg, 0.76 mmol, 98%) as a white solid. $^1$H NMR (300 MHz, $CDCl_3$) δ 6.53 (s, 2H), 4.14 – 3.88 (m, 4H), 3.32 (p, *J* = 6.6, 5.6 Hz, 2H), 3.11 – 2.77 (m, 6H), 2.41 – 2.21 (m, 8H), 1.98 – 1.87 (m, 4H), 1.36 (s, 6H), 1.06 (d, *J* = 6.8 Hz, 6H). $^{13}$C NMR (101 MHz, $CDCl_3$) δ 155.81, 142.29, 141.10, 136.27, 130.66 , 122.35, 111.71, 66.72, 41.92, 39.62, 38.22, 33.29, 20.63, 18.96, 14.52. HRMS (ESI+) calcd. for $[M + H]^+$: 464.3343, found: 464.3353.

(*P,P*)-*cis*-**4**

To a solution of phenyl chloroformate (87.8 µL, 0.70 mmol) in DCM (3 mL) was added (*P*,*P*)-*cis*-**3** (150 mg, 0.32 mmol) and *N*,*N*-diisopropylethylamine (121.9 µL, 0.70 mmol) at 0 °C. After stirring for 16 h at room temperature, the reaction mixture was concentrated *in vacuo*. The crude product was purified by column chromatography ($SiO_2$, EtOAc:pentane = 3:7) to afford compound (*P*,*P*)-*cis*-**4** (135 mg, 0.19 mmol, 60%) as a white solid. $^1H$ NMR (400 MHz, $CDCl_3$) δ 7.33 (dd, *J* = 8.5, 7.3 Hz, 4H), 7.18 (t, *J* = 7.4 Hz, 2H), 7.07 (d, *J* = 7.8 Hz, 4H), 6.53 (s, 2H), 5.56 (t, *J* = 5.7 Hz, 2H), 4.06 – 3.90 (m, 4H), 3.54 – 3.30 (m, 6H), 3.08 (dd, *J* = 14.7, 6.3 Hz, 2H), 2.41 (d, *J* = 14.6 Hz, 2H), 2.27 (s, 6H), 2.00 (p, *J* = 6.2 Hz, 4H), 1.45 (s, 6H), 1.10 (d, *J* = 6.7 Hz, 6H). $^{13}C$ NMR (101 MHz, $CDCl_3$) δ 155.6, 154.8, 151.3, 142.4, 141.1, 136.6, 130.8, 129.3, 125.3, 122.2, 121.8, 111.9, 67.2, 41.9, 39.6, 38.2, 29.5, 20.6, 19.0, 14.6. HRMS (ESI+) calcd. for $[M+H]^+$: 703.3742, found: 703.3733.

(*P*,*P*)-*cis*-**M1**

To a solution of (*P*,*P*)-*cis*-**4** (100.0 mg, 0.14 mmol) in DMSO (3 mL) was added triethylamine (43.2 µL, 0.31 mmol) and compound **5**[2] (119 mg, 0.31 mmol), which was synthesized as described in our previous paper.[2] The mixture was stirred at 60 °C for 16 h. After cooling to room temperature, water (3 mL) was added to the solution, followed by extraction with EtOAc. The organic layer was dried with $Na_2SO_4$ and concentrated *in vacuo*. The resulting viscous oil was dissolved in DCM (2 mL) and the product precipitated by adding pentane (8 mL). The precipitation was repeated three times. After drying *in vacuo*, pure (*P*,*P*)-*cis*-**M1** (76.0 mg, 0.06 mmol, 42%) was obtained as a colorless sticky solid. $^1H$ NMR (500 MHz, $CDCl_3$) δ 6.53 (s, 2H), 5.25 (t, *J* = 5.7 Hz, 2H), 4.99 (t, *J* = 5.5 Hz, 2H), 4.05-3.94 (m, 4H), 3.75 – 3.54 (m, 48H), 3.43 (t, *J* = 6.7 Hz, 4H), 3.32 (tt, *J* = 9.4, 5.1 Hz, 6H), 3.11 (q, *J* = 6.3, 5.5 Hz, 4H), 3.04 (dd, *J* = 14.6, 6.3 Hz, 2H), 2.38 (d, *J* = 14.6 Hz, 2H), 2.23 (s, 6H), 1.99 – 1.87 (m, 4H), 1.55 (p, *J* = 6.5 Hz, 4H), 1.43 (p, *J* = 7.1 Hz, 4H), 1.37 – 1.27 (m, 14H), 1.07 (d, *J* = 6.7 Hz, 6H). $^{13}C$ NMR (151 MHz, $CDCl_3$) δ 159.1, 155.7, 142.3, 141.1, 136.4, 130.8, 122.2, 112.2, 72.8, 71.4, 70.8, 70.7, 70.7, 70.7, 70.7, 70.4, 70.2, 66.8, 61.8, 41.7, 40.4, 38.3, 37.9, 30.4, 30.3, 29.6, 26.8, 26.0, 20.6, 18.9, 14.7. HRMS (ESI+) calcd. for $[M+H]^+$: 1278.8392, found: 1278.8391.

Racemic stable *cis*-**M1** was synthesized through the same method starting from racemic stable *cis*-**1.**

**4. UV-vis spectroscopic study on the rotation of molecular motors.**

The solvents, methanol and water were degassed by purging with argon for 30 min prior to use in the photoisomerization experiments.

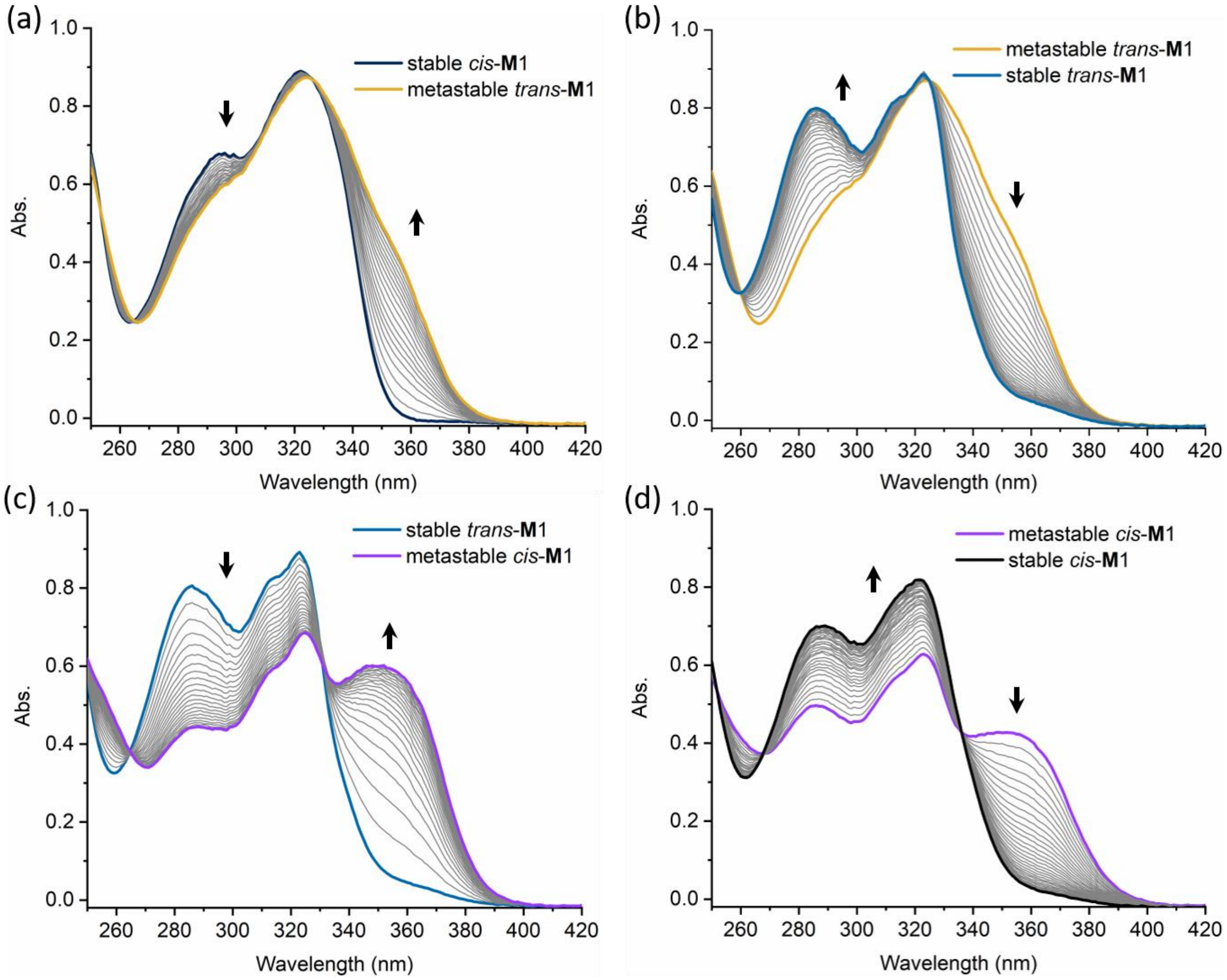


**Figure S2**. UV-vis absorption spectra of stable *cis*-**M1** 39 µM in MeOH (a) upon 312 nm light irradiation for 3.5 min to get metastable *trans*-**M1** at –15 ºC, (b) keeping in the dark at –15 ºC for 37 min to reach stable *trans*-**M1**, (c) subsequent irradiating with 312 nm light at –15 ºC for 35 min to get metastable *cis*-**M1**, (d) finally keeping in the dark at 45 ºC for 5 h to recover stable *cis*-**M1**.

## 5. Eyring analysis on the thermal helix inversion of molecular motors.

The thermal helix inversion studies were performed according to the previous work of our group.[3]

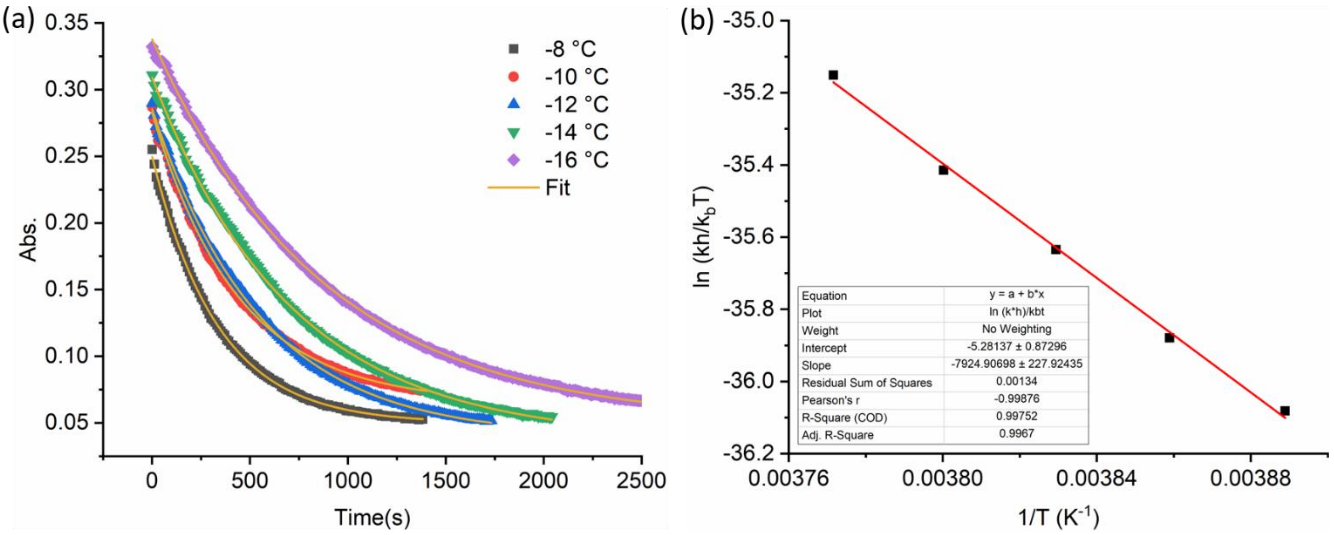


**Figure S3.** (a) Time-dependent absorption changes at 365 nm during the THI of metastable (*M*,*M*)-*trans*-**M1** in MeOH at different temperatures. (b) Eyring analysis on the THI of metastable (*M*,*M*)-*trans*-**M1** in MeOH.

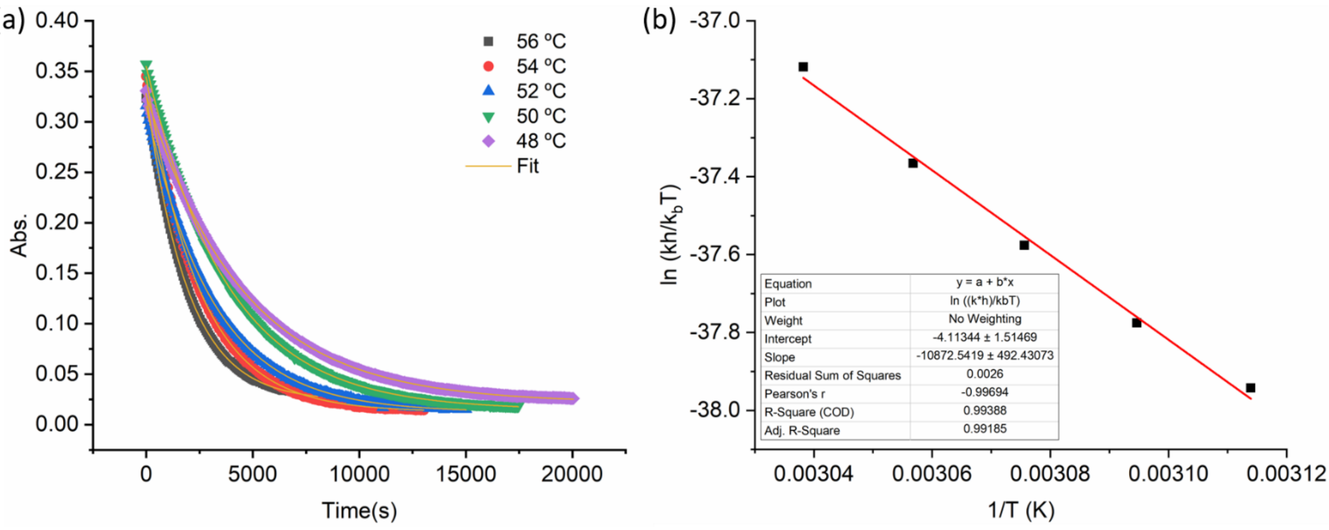


**Figure S4.** (a) Time-dependent absorption changes at 365 nm during the THI of metastable (*M*,*M*)-*cis*-**M1** in MeOH at different temperatures. (b) Eyring analysis on the THI of metastable (*M*,*M*)-*cis*-**M1** in MeOH.

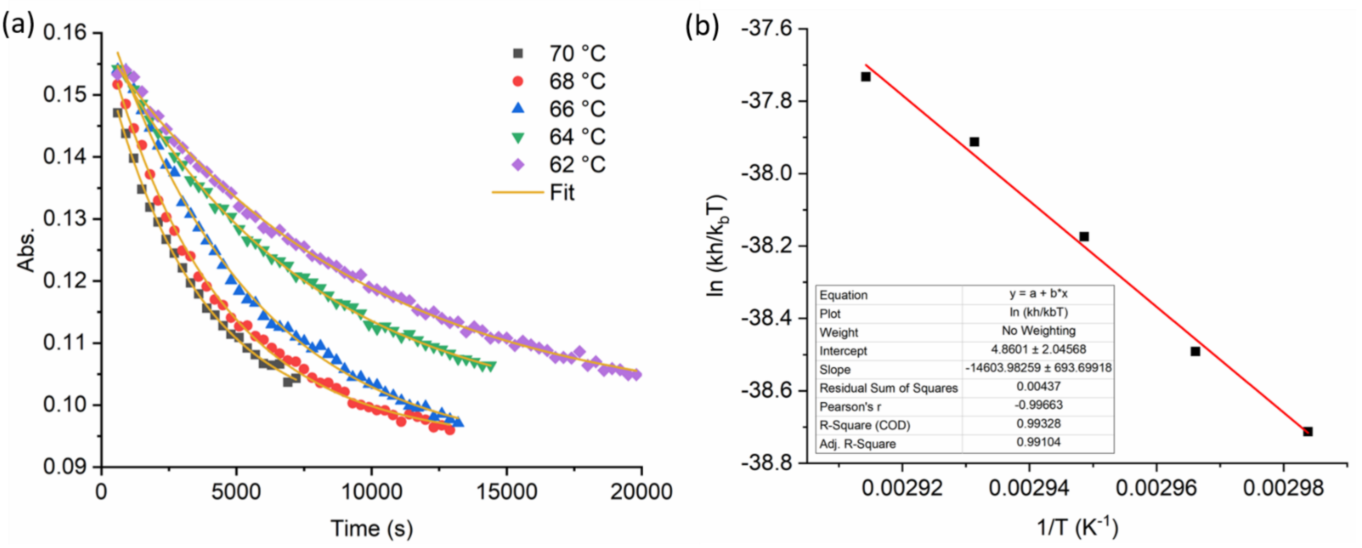


**Figure S5.** (a) Time-dependent absorption changes at 365 nm during the THI of metastable (*M*,*M*)-*cis*-**M1** in water at different temperatures. (b) Eyring analysis on the THI of metastable (*M*,*M*)-*cis*-**M1** in water.

### 6. $^1$H NMR study on the rotation of molecular motors.

Since **M1** form aggregates in water, the $^1$H NMR spectra only shows the broadening of proton resonances (4.0-3.0 ppm) of hexaethylene glycol in $D_2O$ (Figure S6). It is not possible to quantify the ratio of isomers due to the absence of the characteristic signal of molecular motors in $D_2O$. Therefore we measured the ratio of isomers by dissolving the sample in MeOD after removing the water using freeze-drier. The supramolecular structure did not persist in MeOH, evidenced by the $^1$H NMR and DLS measurements. The nearly complete conversion of (*M,M*)-*cis*-**M1** to (*P,P*)-*cis*-**M1** after THI was demonstrated clearly by the disappearance of the signals (*M,M*)-*cis*-**M1** in $^1$H NMR spectra (Figure S7d), while the (*P,P*)-*trans*-**M1** are still present because they did not undergo the THI (Figure S7c, d). The result from the NMR study is consistent with the CD study.

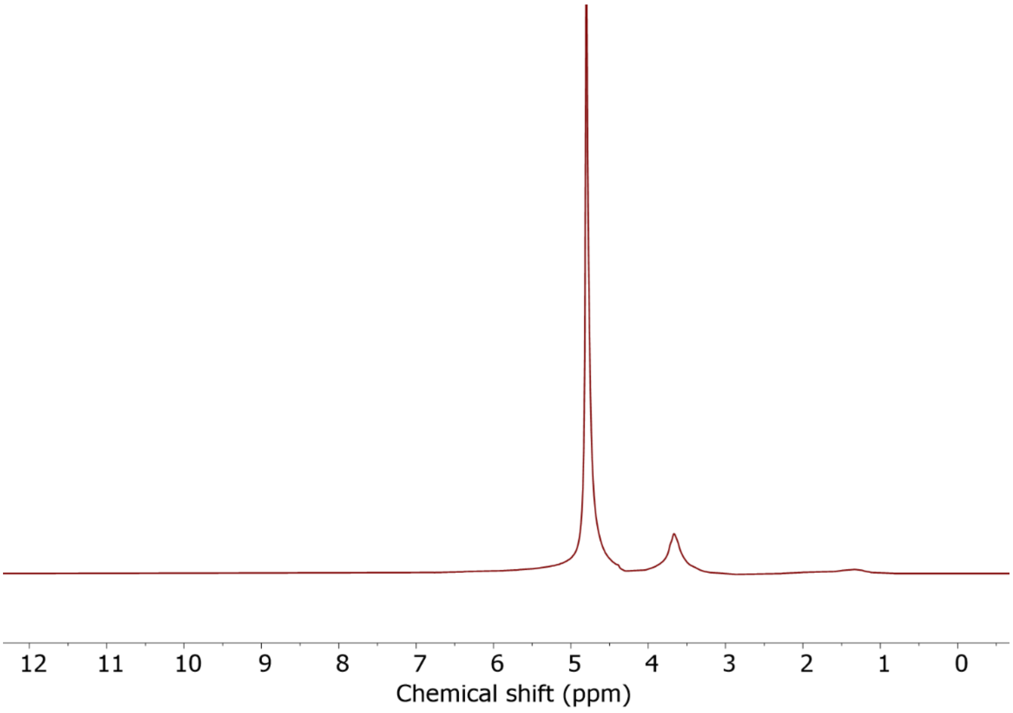


**Figure S6**. $^1$H NMR spectra ($D_2O$, 298 K, 500 MHz) of (*P,P*)-*cis*-**M1** (780 µM).

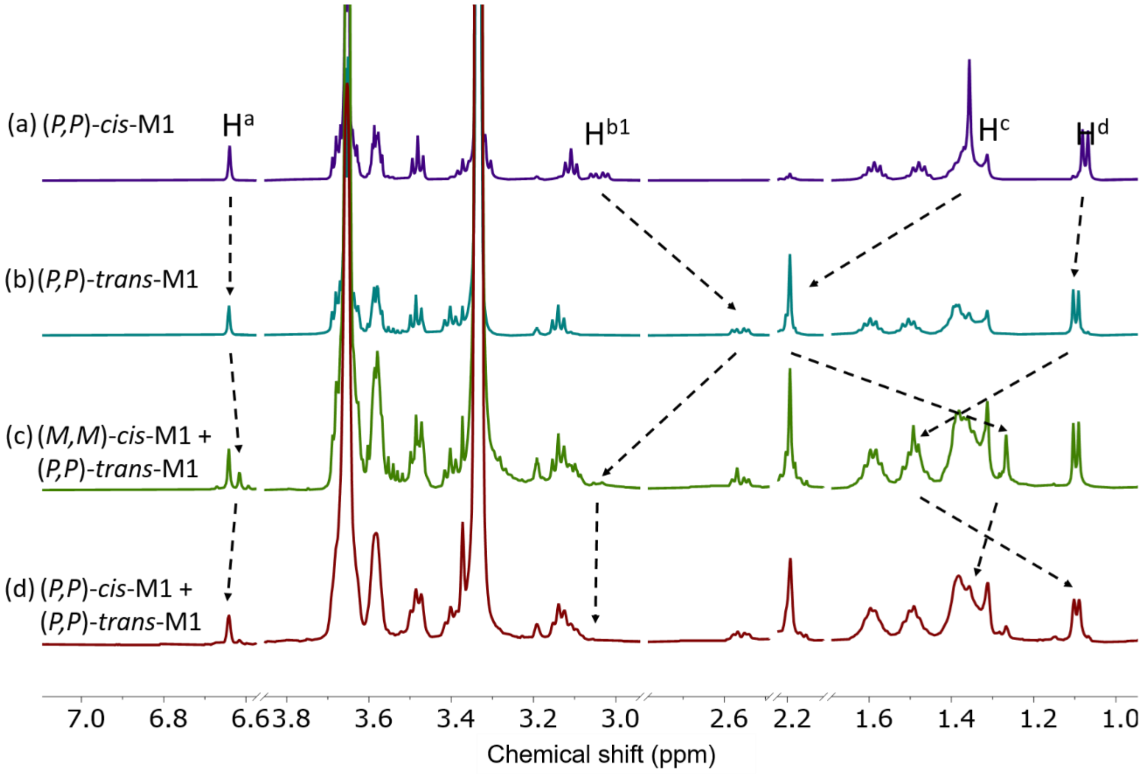


**Figure S7.** $^{1}$H NMR spectra ($CD_3OD$, 298 K, 500 MHz) of (a) stable (*P*,*P*)-*cis*-**M1**, (b) after irradiation with 312 nm light for 2 min and subsequently keeping in the dark at room temperature for 10 min in water to get stable (*P*,*P*)-*trans*-**M1**, (c) followed by irradiating with 312 nm light for 10 min to reach a PSS mixture of stable (*P*,*P*)-*trans*-**M1** and metastable (*M*,*M*)-*cis*-**M1** with a ratio of 68:32, (d) finally keeping in the dark at 45 ºC for 3 d to achieve the THI of metastable (*M*,*M*)-*cis*-**M1** to stable (*P*,*P*)-*cis*-**M1.** Samples were characterized after removal of water by freeze-drying and dissolving in $CD_3OD$.

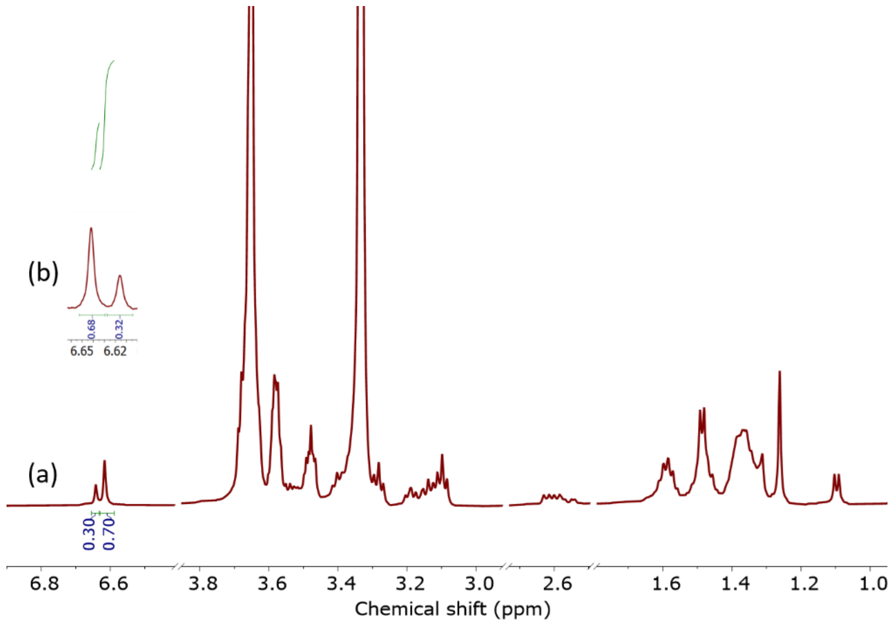


**Figure S8.** (a) $^{1}$H NMR spectra ($CD_3OD$, 298 K, 500 MHz) of stable (*P*,*P*)-*cis*-**M1** after irradiation with 312 nm light for 2 min and subsequently keeping in the dark at room temperature for 10 min in water, followed by irradiating with 312 nm light for 20 min in a water/THF (7/3) solution to reach a PSS mixture of stable *trans*-**M1** and metastable *cis*-**M1** (30:70). The sample was characterized after drying from water/THF mixture and dissolving in $CD_3OD$; (b) integration of signal of stable (*P*,*P*)-*trans*-**M1** and metastable (*M*,*M*)-*cis*-**M1** shown in Figure S7c**.**

## 7. Fourier transform infrared spectroscopy (FTIR).

Supramolecular polymers of stable (*P*,*P*)-*cis*-**M1** and stable (*P*,*P*)-*trans*-**M1** were measured on a PerkinElmer Spectrum 400. Samples were characterized after freeze-drying from water.

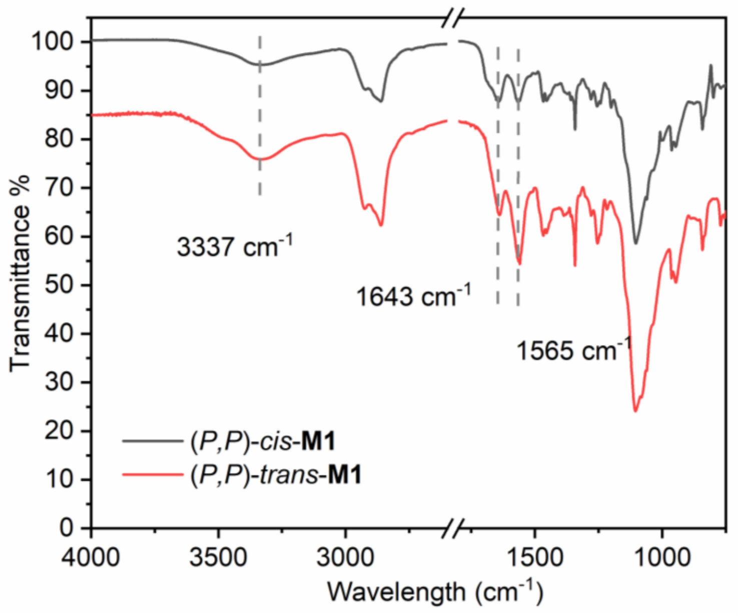


**Figure S9.** FTIR spectra of stable (*P*,*P*)-*cis*-**M1** and stable (*P*,*P*)-*trans*-**M1**. Samples were characterized after freeze-drying from water.

## 8. Linear dichroism (LD) study.

To rule out the potential CD artifacts, we measured LD together with CD and absorption spectra of the same sample (Figure S10). No linear dichroism (LD) signal was measured for the sample, indicating that the CD signals are induced from molecules to supramolecular polymers.

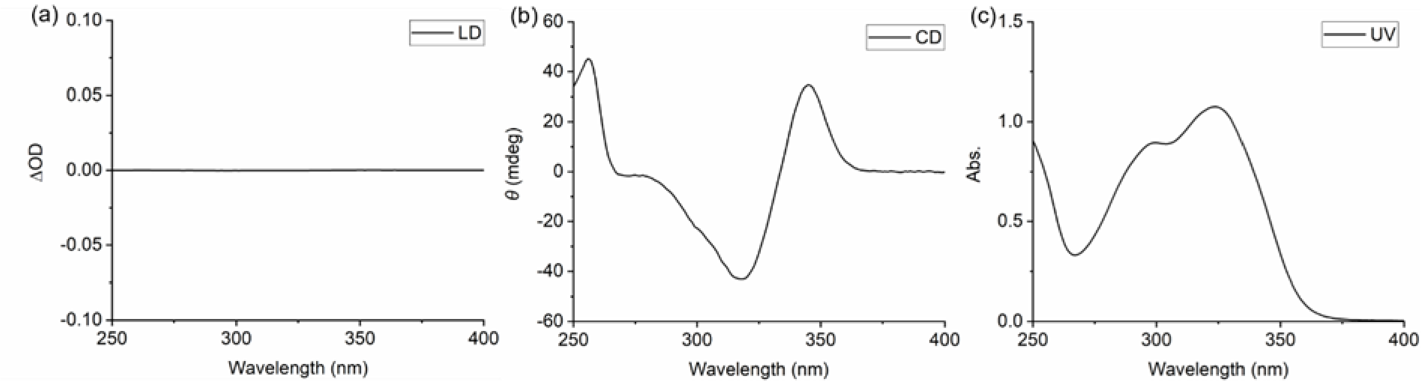


**Figure S10.** (a) LD, (b) CD, and (C) absorption spectra of supramolecular polymers formed by (*P*,*P*)-*cis*-**M1** (650 μM) in water.

## 9. Temperature-dependent dynamic light scattering (DLS) measurements

In order to investigate the polymerization mechanism, we monitored the molar ellipticity at 310 nm relative to the formation of helical aggregates upon heating the water solution of (*P*,*P*)-*cis*-**M1** from 293 K to 353 K (Figure S11a). Increasing the temperature, the CD signal for helical aggregates decreased gradually, suggesting an increase in the disorder of the helical packing of the molecules. However, the temperature-

dependent degree of aggregation, $\alpha_{agg}$, shows deviation with the fits using both the isodesmic and the cooperative models (Figure S11b).[4,5] To figure out the reason for this phenomenon, we also monitored the heating process by dynamic light scattering (DLS) (Figure S12). The size of the assemblies first decreased with the increasing temperature, suggesting the disassembly of the helical fibers. However, the hydrodynamic diameter of the assembly sharply increased to a range of 100-1000 nm when the temperature reached 60 °C. The larger assemblies at high temperatures are possible due to the breaking of the hydrogen bonding between the hexaethylene glycol chains and water when above the low critical solution temperature (LCST), resulting in phase separation. The LCST-Type phase separation can be found in the aqueous solution of covalent polymers and supramolecular assemblies that functioned with poly(ethylene glycol) or oligo(ethylene glycol) moieties.[6–8] The additional phase transition in the healing process of our molecules is distinct from the melting process of supramolecular polymers in organic solvents; therefore, it is difficult to fit with the present model and get thermodynamic parameters. After cooling to 20 °C and stabilizing for 2 hours, the original size was obtained again, suggesting the reformation of the supramolecular polymers.

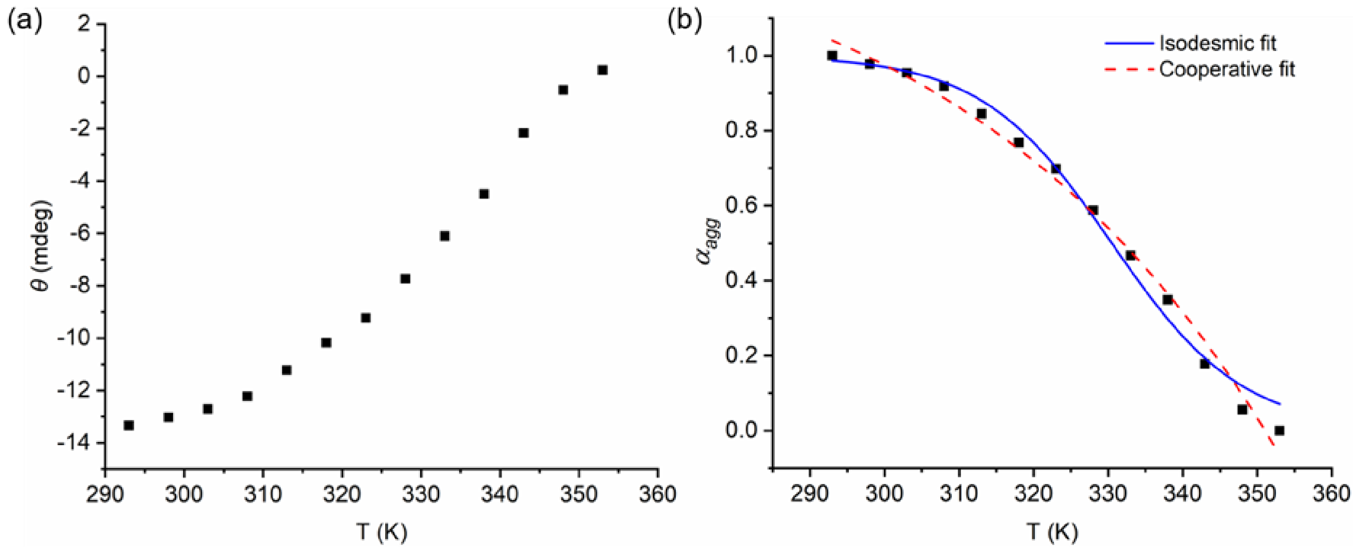


**Figure S11.** (a) Temperature-dependent molar ellipticity ($\Theta$) at 310 nm upon heating an aqueous solution of the supramolecular polymer formed by (*P*,*P*)-*cis*-**M1** (52 μM) from 293 K to 353 K, (b) Temperature-dependent degree of aggregation, $\alpha_{agg}$ of (*P*,*P*)-*cis*-**M1** in water estimated from CD spectra. The curves show fits calculated according to the isodesmic (blue) and cooperative (red) models.

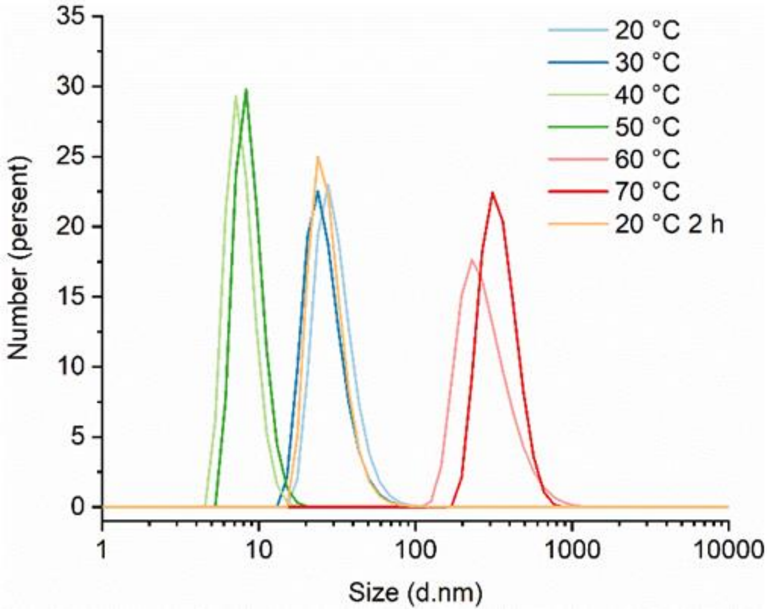


**Figure S12**. DLS profile of (*P*,*P*)-*cis*-**M1** (52 µM) upon heating to 70 °C and subsequent cooling to 20 C and aging for 2 h.

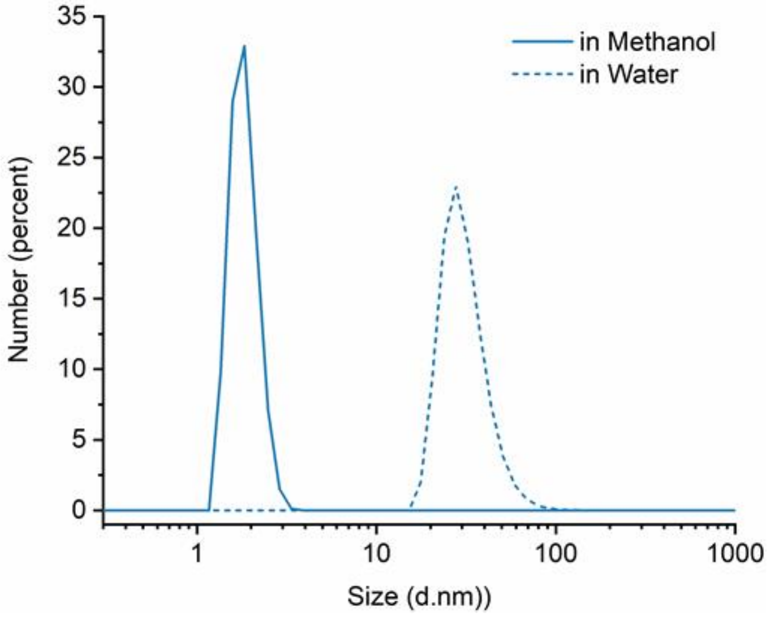


**Figure S13**. DLS profile of (*P*,*P*)-*cis*-**M1** (780 µM) in methanol (solid line) comparing with (*P*,*P*)-*cis*-**M1** (52 µM) in water at 20 °C. The hydrodynamic diameter ($D_h$) of molecular motors is around 2 nm in MeOH, while $D_h$ of aggregates in water is in the range of 20-80 nm at 20 °C.

## 10. Cryo-TEM study.

A water solution of (*P*,*P*)-*cis*-**M1** (1mg/mL) was treated with ultrasound to be dispersed and kept in the dark at room temperature (RT) for 1 h (Figure 5a, S14). All the irradiation process the following protocol using a Spectroline hand-held UV lamp (λ = 312 nm) positioned at a distance of 2 cm from the sample. The sample was first irradiated with 312 nm light for 2 min and kept in the dark at RT for 10 min to get (*P*,*P*)-*trans*-**M1** in a quartz cuvette with 1 mm path length (Figure 5b, S15). Then the sample was irradiated with 312 nm light for 10 min to get a mixture of metastable (*M*,*M*)-*cis*-**M1** and stable (*P*,*P*)-*trans*-**M1** with a ratio of 32:68

(Figure 5c, S16). Finally, the sample was kept in the dark at 45 ºC for 3 d to get a mixture of stable (*P,P*)-*cis*-**M1** and stable (*P,P*)-*trans*-**M1** (Figure S17). A water solution of (*P,P*)-*trans*-**M1** was prepared using the above protocol starting from the (*P,P*)-*cis*-**M1**. After adding THF to the sample to form a water/THF(7/3) solution, it was irradiated for 20 min to get a mixture of metastable (*M,M*)-*cis*-**M1** and stable (*P,P*)-*trans*-**M1** (70:30). Then the sample was kept in the dark at 45 ºC for 5 h to get a mixture of (*P,P*)-*cis*-**M1** and (*P,P*)-*trans*-**M1**. The cryo-TEM images were measured after removal of the water/THF solvent and preparation in water again (Figure 5d, S19) to make a rigorous comparison with the above images (Figure 5a-c, S14-17). The ratio of isomers was determined by integrating the NMR signals (Figure S8). A few microliters of each sample solution were placed on holey carbon-coated copper grids (Quantifoil 3.5/1, Quantifoil Micro Tools, Jena, Germany). Grids with samples were vitrified in liquid nitrogen (Vitrobot, FEI, Eindhoven, The Netherlands) and transferred to a FEI Talos Arctica cryo-electron microscope operating at 200 keV with a postcolumn energy filter (Gatan) in zero-loss mode, with a 20-eV slit.

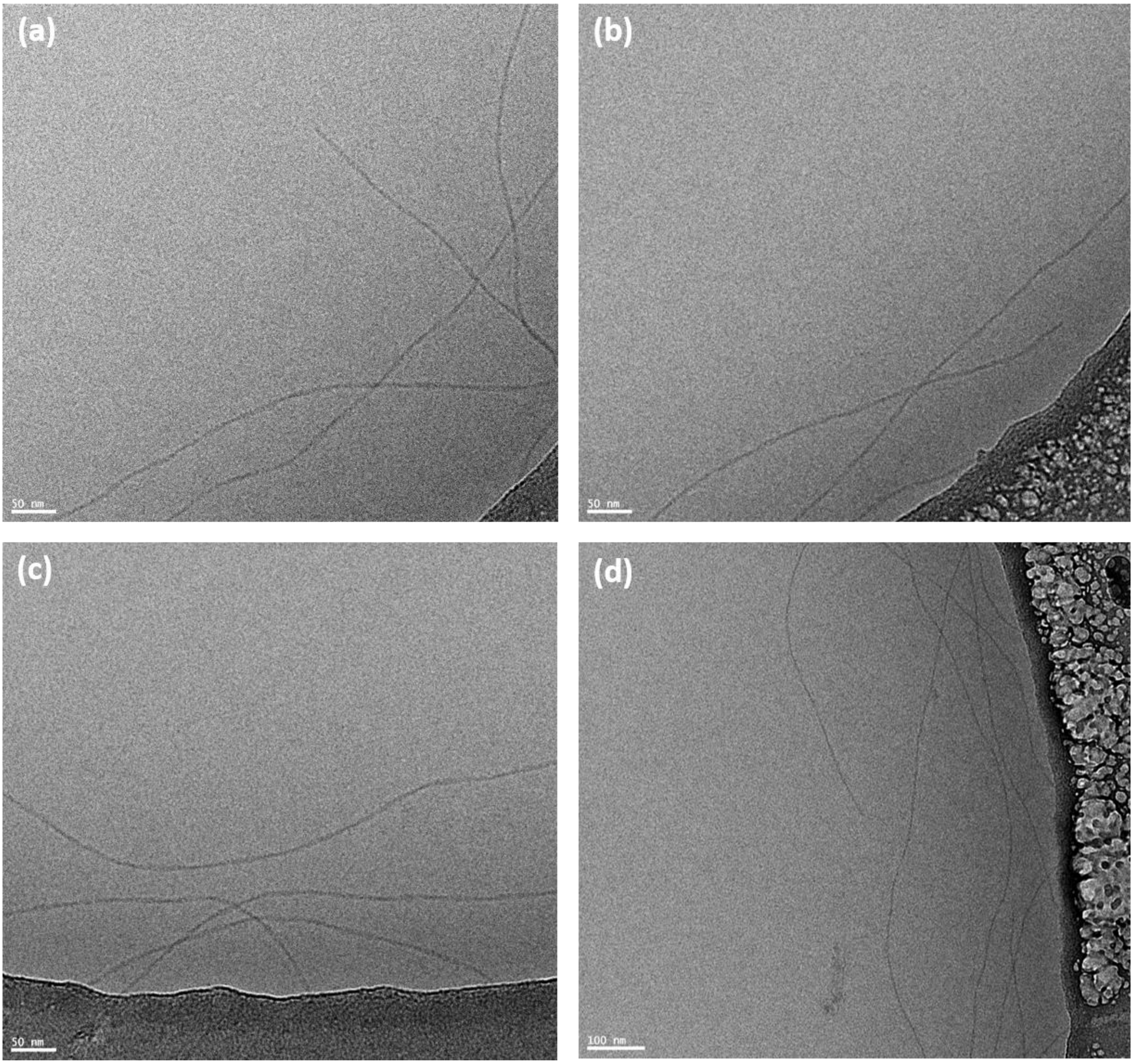


**Figure S14**. Cryo-TEM images of (*P,P*)-*cis*-**M1** (780 μM) in water, showing the helical fibers.

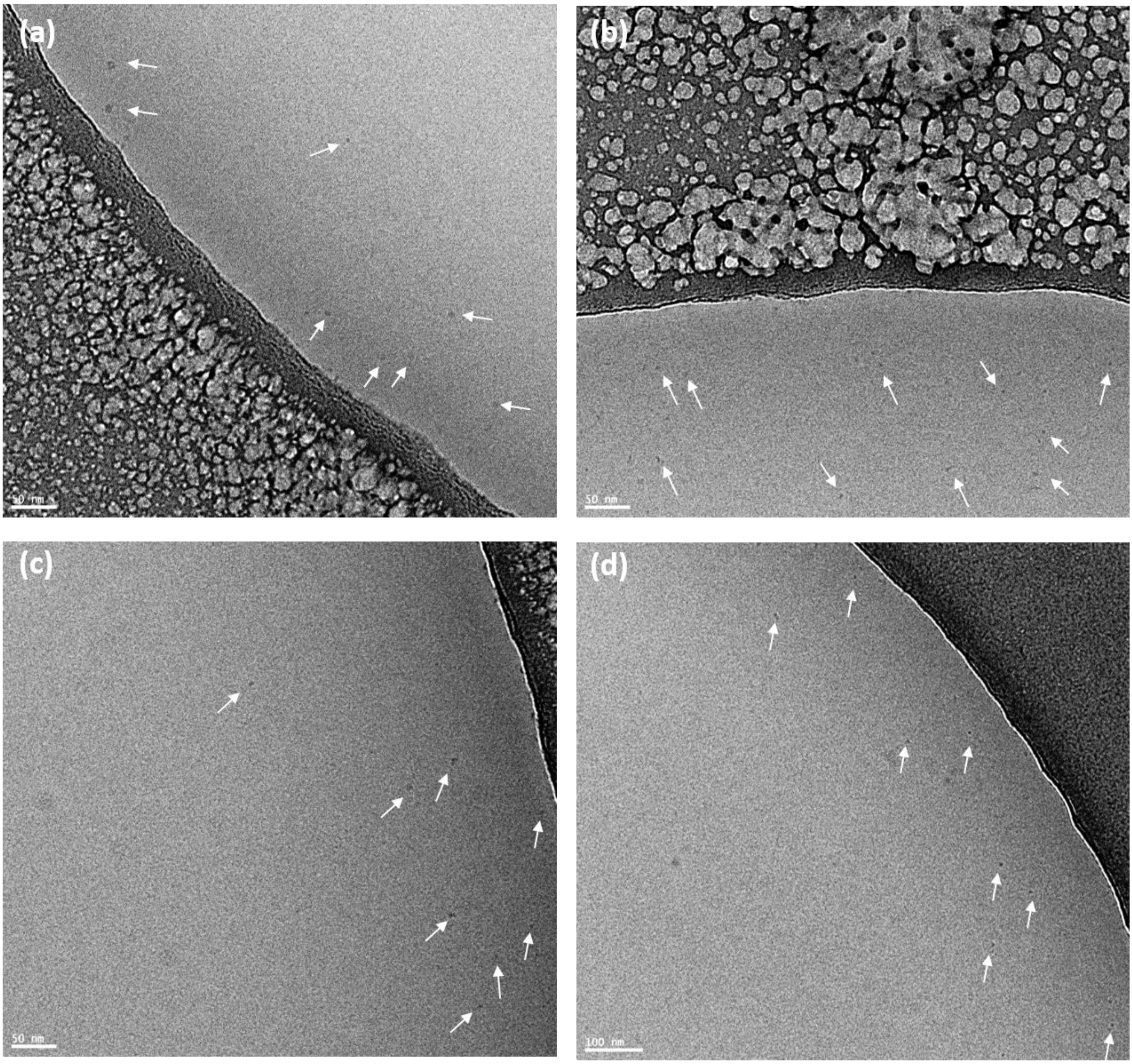


**Figure S15**. Cryo-TEM images of (*P,P*)-*trans*-**M1**, obtained by irradiating (*P,P*)-*cis*-**M1** (780 µM) with 312 nm light for 2 min and keeping in the dark for 10 min in water. Micelles are pointed out with arrows for clearance, and not all are pointed. Similar morphology aggregates without white arrows are also micelles.

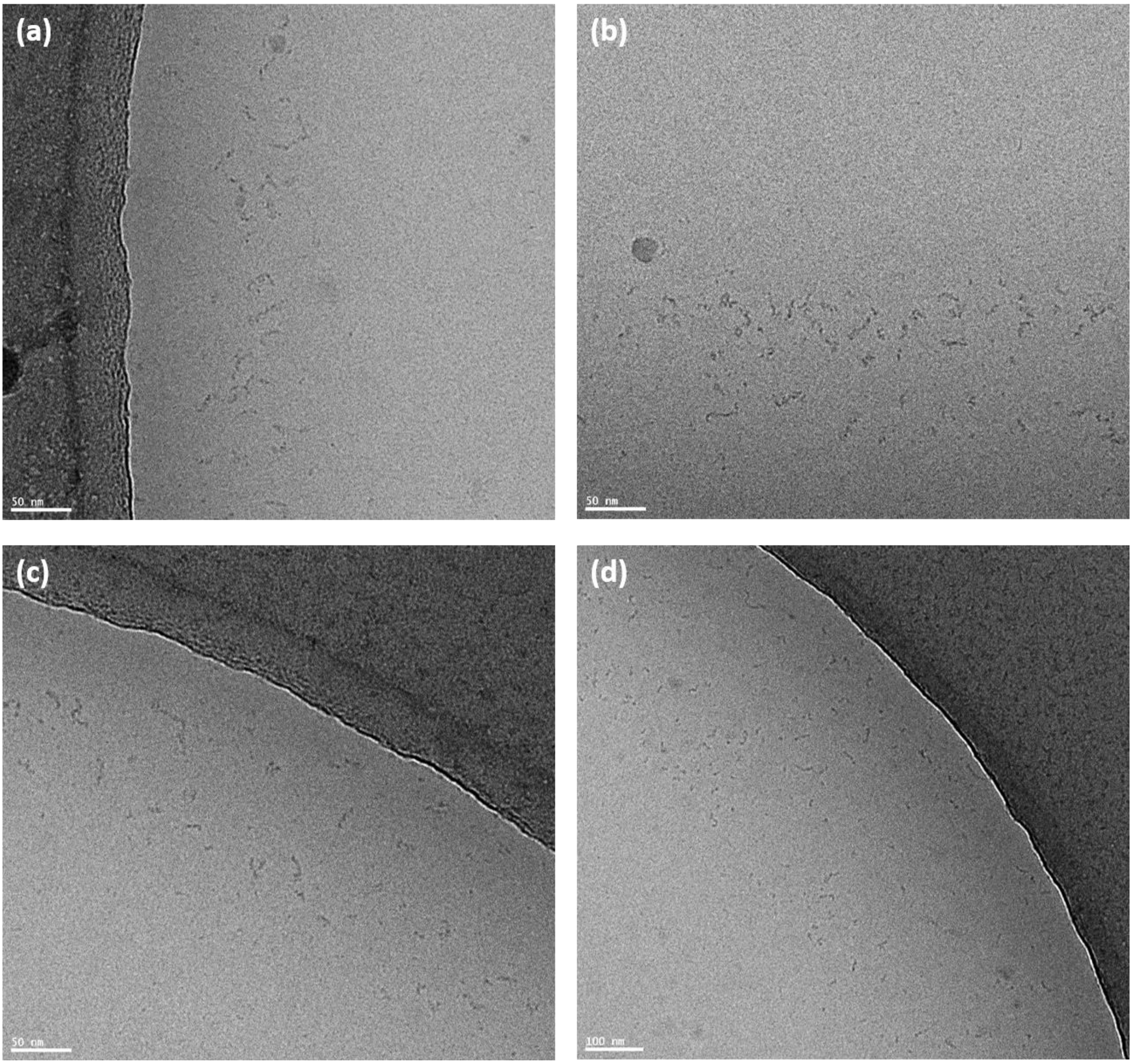


**Figure S16**. Cryo-TEM images of a PSS mixture of (*M,M*)-*cis*-**M1** and (*P,P*)-*trans*-**M1**, obtained by irradiating (*P,P*)-*cis*-**M1** (780 μM) with 312 nm light for 2 min and keeping in the dark for 10 min in water, followed by subsequent irradiation with 312 nm light for 10 min in water, showing the worm-like micelles.

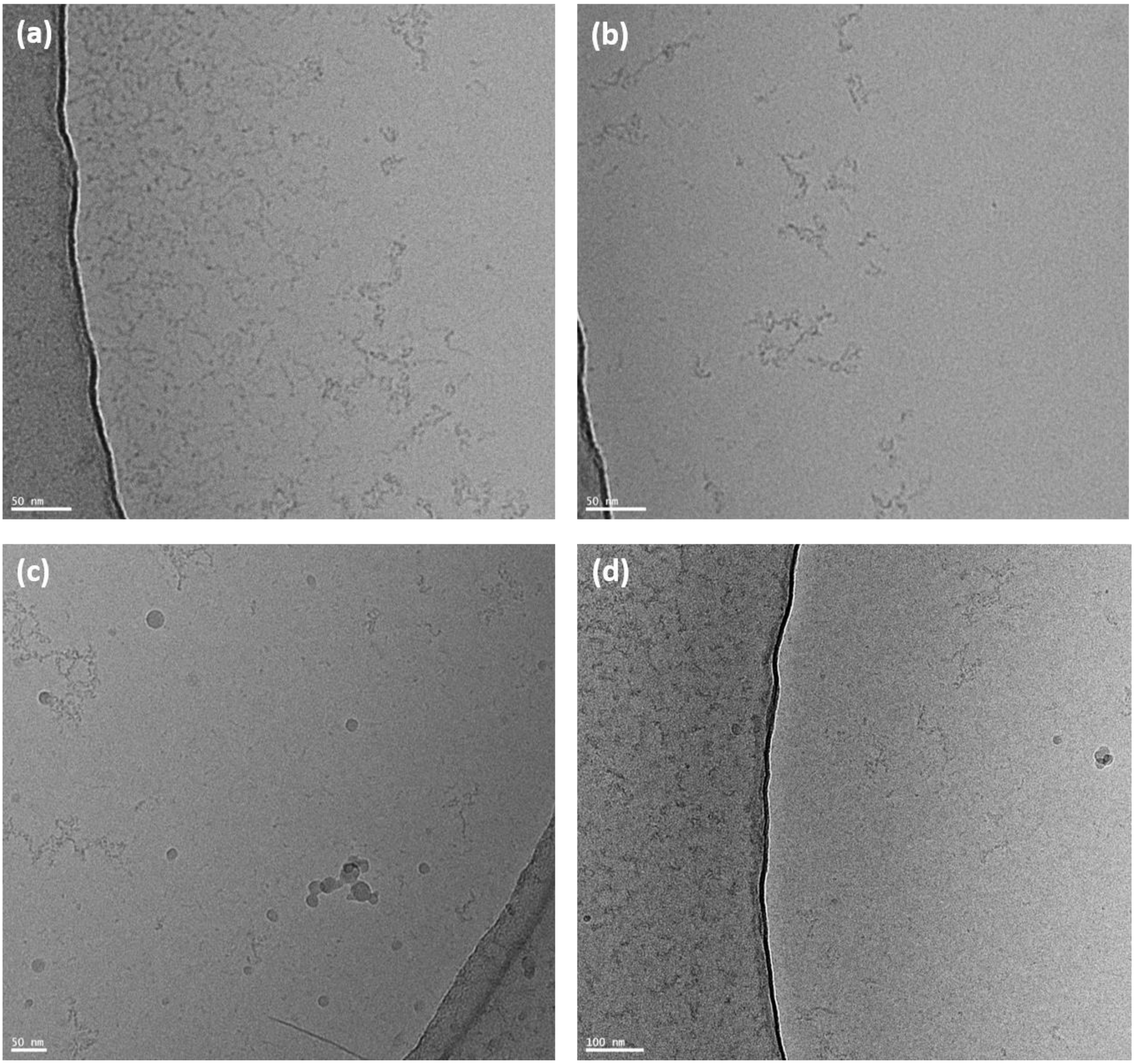


**Figure S17**. Cryo-TEM images of a mixture of stable (*P,P*)-*cis*-**M1** and stable (*P,P*)-*trans*-**M1**, obtained by warming the above PSS mixture of (*M,M*)-*cis*-**M1** and (*P,P*)-*trans*-**M1** at 45 ºC for 3 d in water, showing the worm-like micelles.

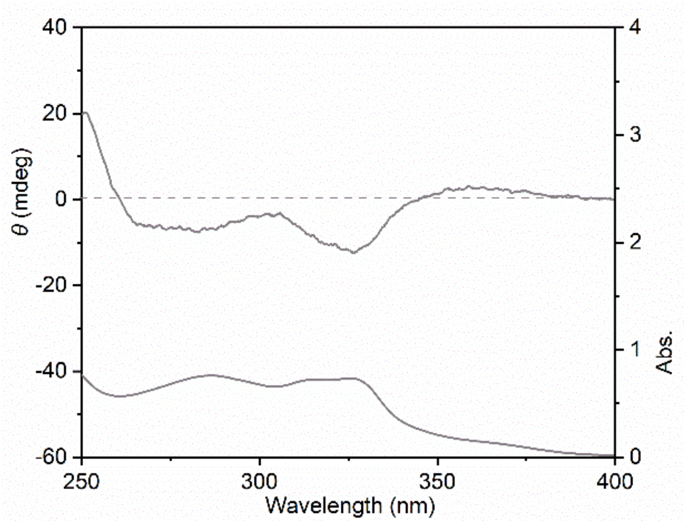


**Figure S18**. CD and UV-vis absorption spectra of the identical sample in the Cryo-TEM image Figure S17 (a mixture of stable (*P*,*P*)-*cis*-**M1** and stable (*P*,*P*)-*trans*-**M1** in water).

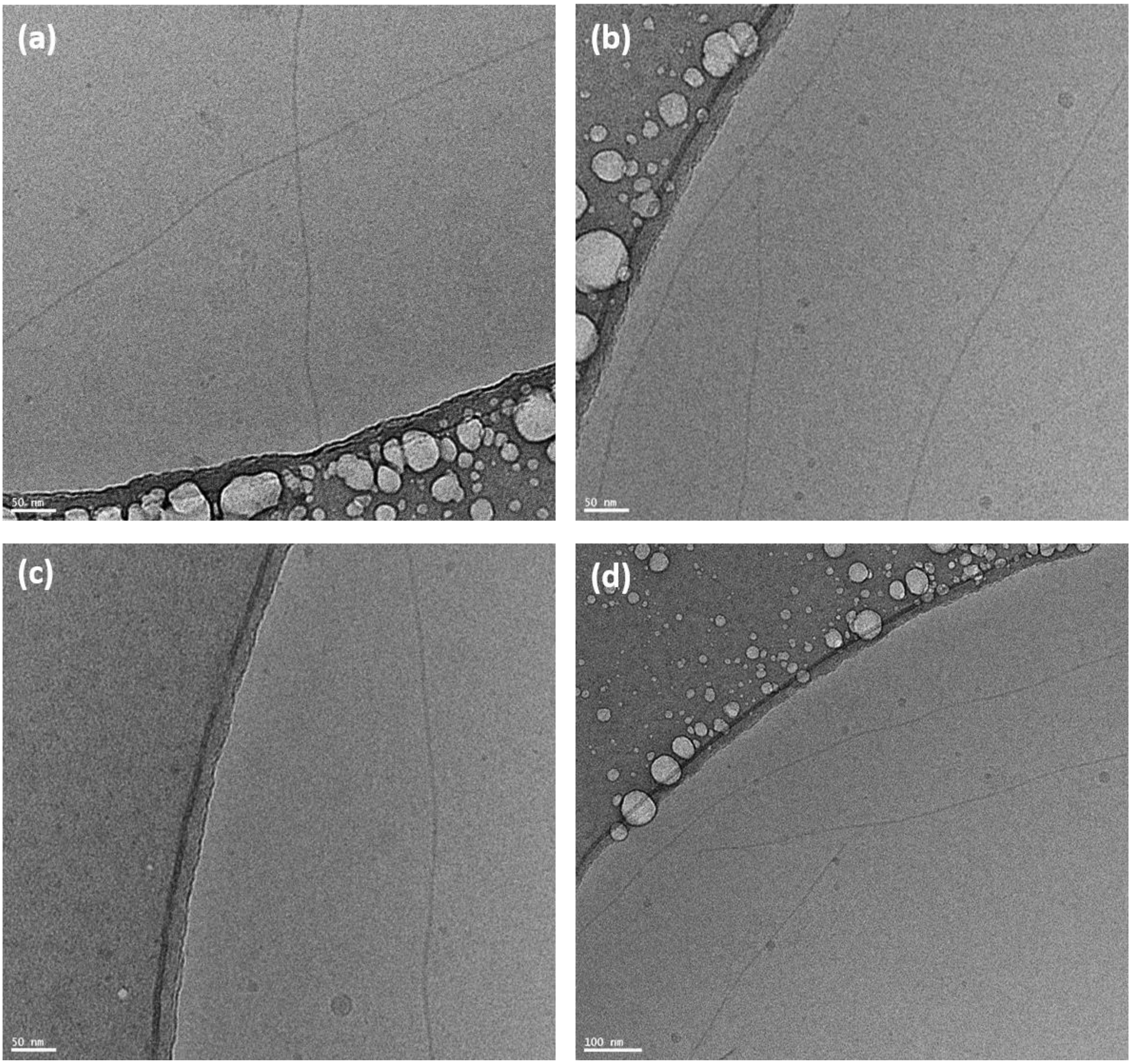

**Figure S19**. Cryo-TEM images of recovered helical fibers of (*P,P*)-*cis*-**M1**, obtained by irradiating (*P,P*)-*cis*-**M1** (780 μM) with 312 nm light for 2 min and keeping in the dark for 10 min in water, followed by subsequent irradiation with 312 nm light for 20 min and warming 45 ºC for 5 h in a water/THF mixture. The cryo-TEM images were measured after removal of the water/THF solvent and preparation in water again.

## 11. SAXS measurements.

Small-angle X-ray scattering measurements were performed using the Multipurpose Instrument for Nanostructure Analysis (MINA) diffractometer at the University of Groningen equipped with Cu rotating anode (λ=1.5413Å). The sample to detector distance was 114.95 cm, and the SAXS patterns were collected using a Bruker Vantec 500 detector with a pixel size of 0.136 mm. The direct beam position and the scattering angle scale were calibrated using a standard silver behenate powder. A water solution of (*P,P*)-*cis*-**M1** (1mg/ml) was added in a quartz capillary with an average diameter of 2 mm sealed with capillary wax to avoid evaporation. The sample was placed in the vacuum chamber of the X-ray machine in order to remove the contribution coming from air absorption and scattering. The temperature inside the chamber was stabilized to 25 °C. SAXS 1D profiles were obtained by radially averaging the scattering intensity around the origin of the image (direct beam position) using the Fit2D program. After radial averaging, the scattering contribution from the water and the quartz capillary was subtracted to the experimentally measured data to retrieve the scattering from the aggregates. Irradiation of the sample was conducted inside a quartz capillary using a Spectroline hand-held UV lamp (λ =312nm) positioned at a distance of 2 cm from the sample. The irradiation process is the same as the above protocol for cryo-TEM samples.

SAXS data for the samples (*P,P*)-*cis*-**M1** and PSS mixture of (*M,M*)-*cis*-**M1** and (*P,P*)-*trans*-**M1** in water showing elongated flexible structures, a worm-like model derived from Kholodenko was used.[9] The Kholodenko model allows the determination of the flexible rod cross-sectional radius, the Kuhn length of the object, which is 2 times the length of the rigid section of the flexible object, and eventually the total length of the object.[10,11] In both cases, the total length of the aggregates seems to be larger than the resolution of the SAXS measurements (> 60 nm), and it was kept fixed during the fitting. The Kuhn length of the (*P,P*)-*cis*-**M1** is also > 60nm. For the PSS mixture of (*M,M*)-*cis*-**M1** and (*P,P*)-*trans*-**M1**, the Kuhn length is within the resolution limit and measurable, resulting in a value of 20 nm. For the (*P,P*)-*trans*-**M1** sample, the form factor of a sphere was used instead.[12] The software package SASfit was used to accomplish the fitting.[13] We refer to the given references and the SASfit manual for the complete equations used.

## 12. Fluorescence spectra.

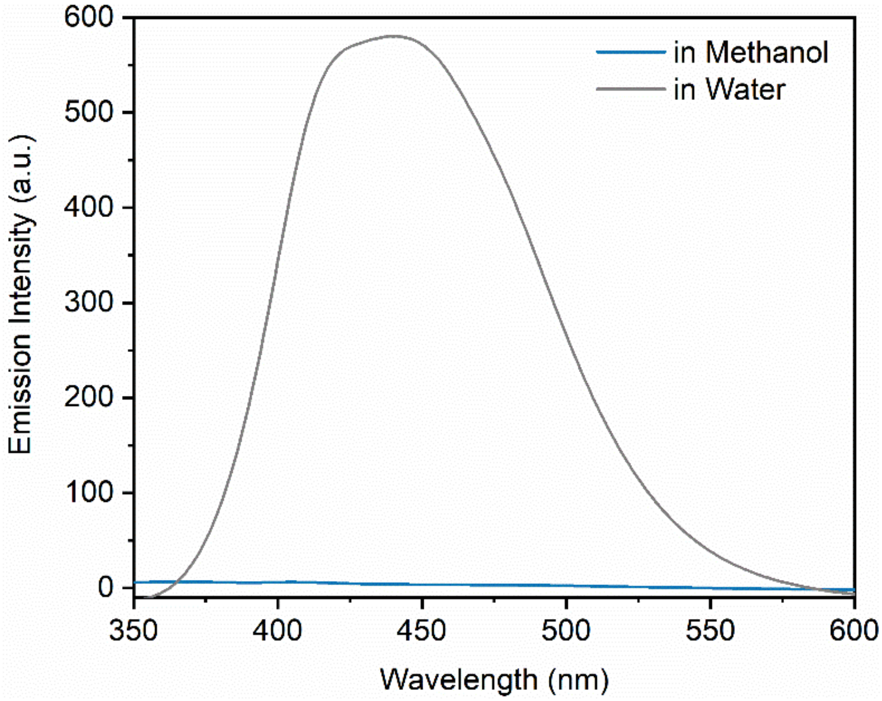


**Figure S20**. Fluorescence spectra of (*P*,*P*)-*cis*-**M1** (78 μM) in MeOH (blue line) and water (gray line), respectively.

## 13. Concentration-dependent emission.

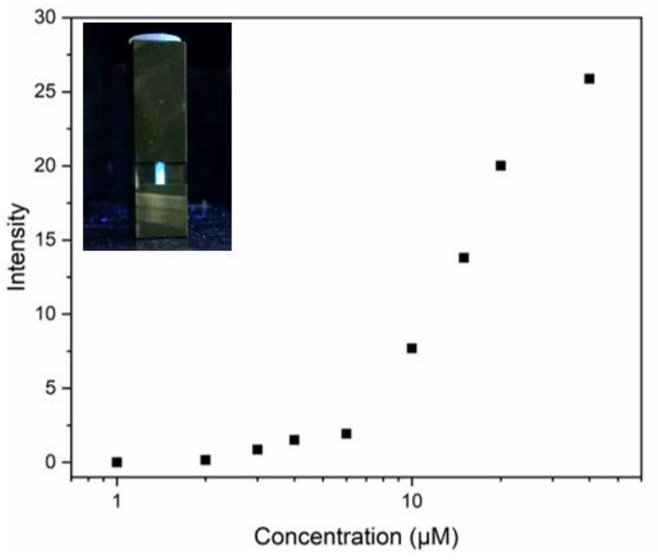


**Figure S21**. Concentration-dependent emission intensity at 455 nm of (*P*,*P*)-*cis*-**M1** in water, $\lambda_{ex}$ = 312 nm. (transparent channel in the cuvette: 1×10 mm)

## 14. Fluorescence quantum yield.

According to the previous study in our group, the rotation of stable *cis*- and *trans*-1$^{st}$ generation motor were activated upon 312 nm irradiation, while the back-switching of metastable *cis* and *trans*-isomer could be induced by exposing them to 365 nm light.[14–16] To avoid the photoisomerization during the fluorescence quantum yield measurements, we used 365 nm as the exciting wavelength for stable (*P*,*P*)-*cis* and (*P*,*P*)-*trans*-**M1**, although (*P*,*P*)-*cis* and (*P*,*P*)-*trans*-**M1** showed relatively lower emission intensity than them under $\lambda_{ex}$

=312. We used 312 nm as the exciting wavelength for metastable (*M*,*M*)-*trans* and (*M*,*M*)-*cis*-**M1**, although these isomers exhibit lower emission intensity than them under 365 nm excitation.

**Table S1.** Fluorescence quantum yield $\Phi_{em}$ of **M1** (78 µM) in water at four states.

| $\lambda_{ex}$ nm | $\Phi_{em}$ (%) | | | |
|---|---|---|---|---|
| | (*P*,*P*)-*cis* | (*M*,*M*)-*trans*[1] | (*P*,*P*)-*trans* | (*M*,*M*)-*cis*[2] |
| **312** | - | 3.2 | - | 1.4 |
| **365** | 9.2 | - | 3.4 | - |

[1,2]obtained by irradiating the (*P*,*P*)-*cis*-**M1** to a PSS mixture.

## 15. Emission lifetime measurements

We measured the emission lifetimes of the supramolecular polymers formed by (*P*,*P*)-*cis*-**M1**, (*P*,*P*)-*trans*-**M1**, and (*M*,*M*)-*cis*-**M1** (PSS mixture). The values we obtained are 0.40 ns, 0.40 ns, and 0.15 ns, respectively, which are close to the resolution of the instrument (Picoquant Picoharp300 with a TAU-SPAD-100 SPC detector with DSN 102-C power supply and a PDL 800-B laser driver with PLS 365 LED). Emission decay was analyzed using the instrument response function obtained with LUDOX scattering solution using Fluorfit software. The fluorescence decay curves are shown in Figure S22-24. It is not possible to measure the emission lifetimes for the (*M*,*M*)-*trans*-**M1** due to the short half-life (2.6 min) for the THI. The fast fluorescence decay is consistent with the competitive photochemical isomerization pathways in aggregates. It should be noted that in the non-aggregated state the compounds do not show emission; note the absence of fluorescence in MeOH solution (Figure S20), underlining that the emission is only observed when the motors are constrained in the supramolecular aggregates.

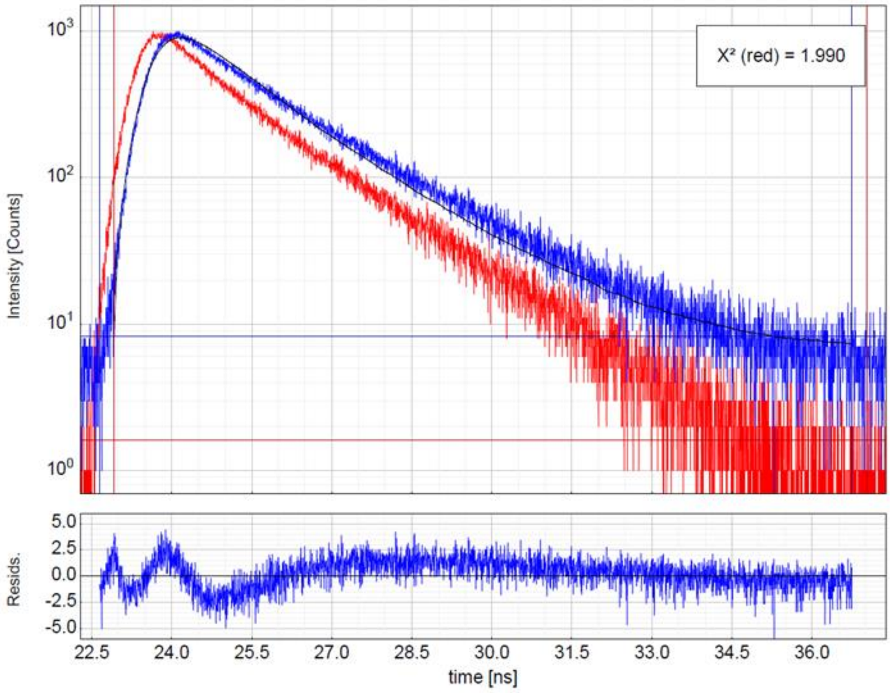


**Figure S22**. The fluorescence decay (blue) of (*P*,*P*)-*cis*-**M1** with the fitted decay and residuals (78 µM in water) and the instrument response curve (red).

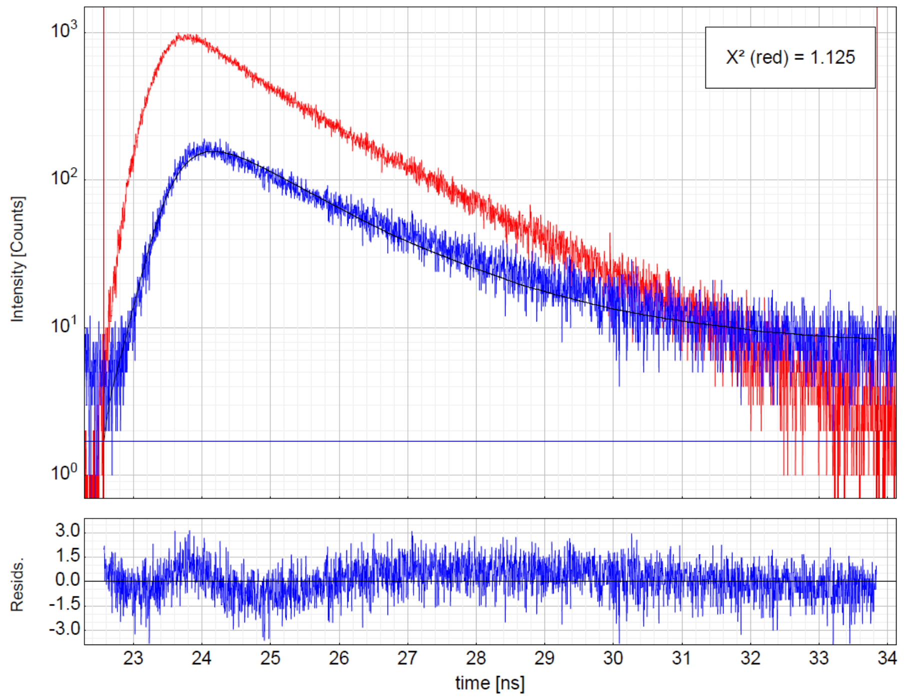


**Figure S23**. The fluorescence decay (blue) of (*P*,*P*)-*trans*-M1 with the fitted decay and residuals (78 µM in water) and the instrument response curve (red).

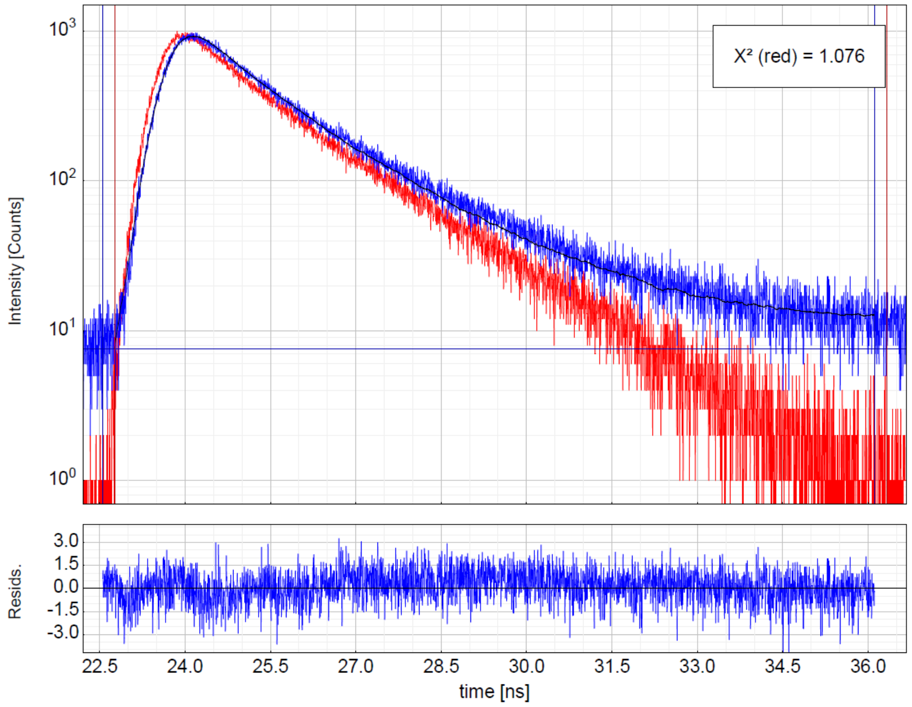


**Figure S24**. The fluorescence decay (blue) of (*M*,*M*)-*cis*-**M1** (PSS mixture) with the fitted decay and residuals (78 µM in water) and the instrument response curve (red).

## 16. NMR and HRMS data.

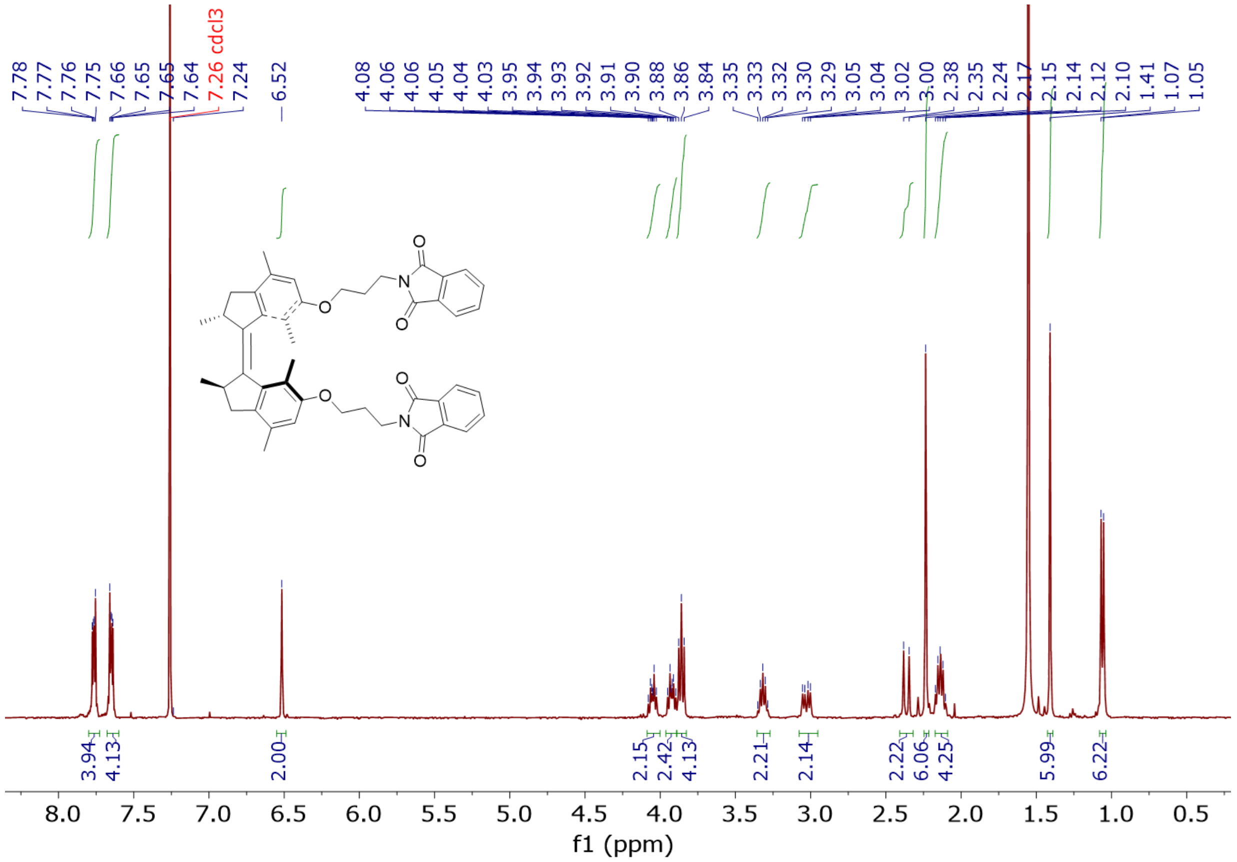

**Figure S25**. $^{1}$H NMR spectrum of (*P*,*P*)-*cis*-**2** ($CDCl_3$, 25 °C, 400 MHz).

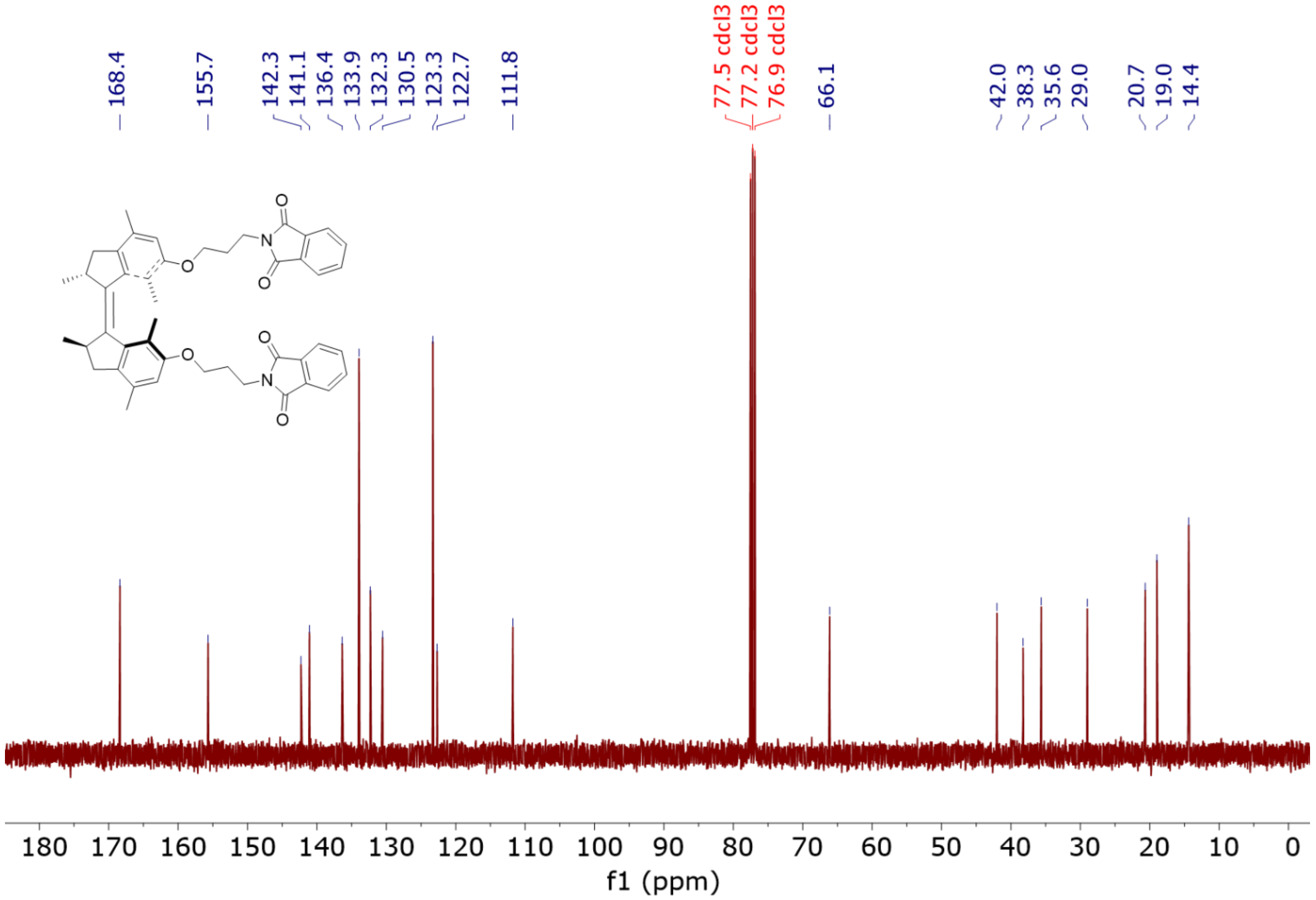


**Figure S26**. $^{13}$C NMR spectrum of (*P*,*P*)-*cis*-**2** ($CDCl_3$, 25 °C, 101 MHz).

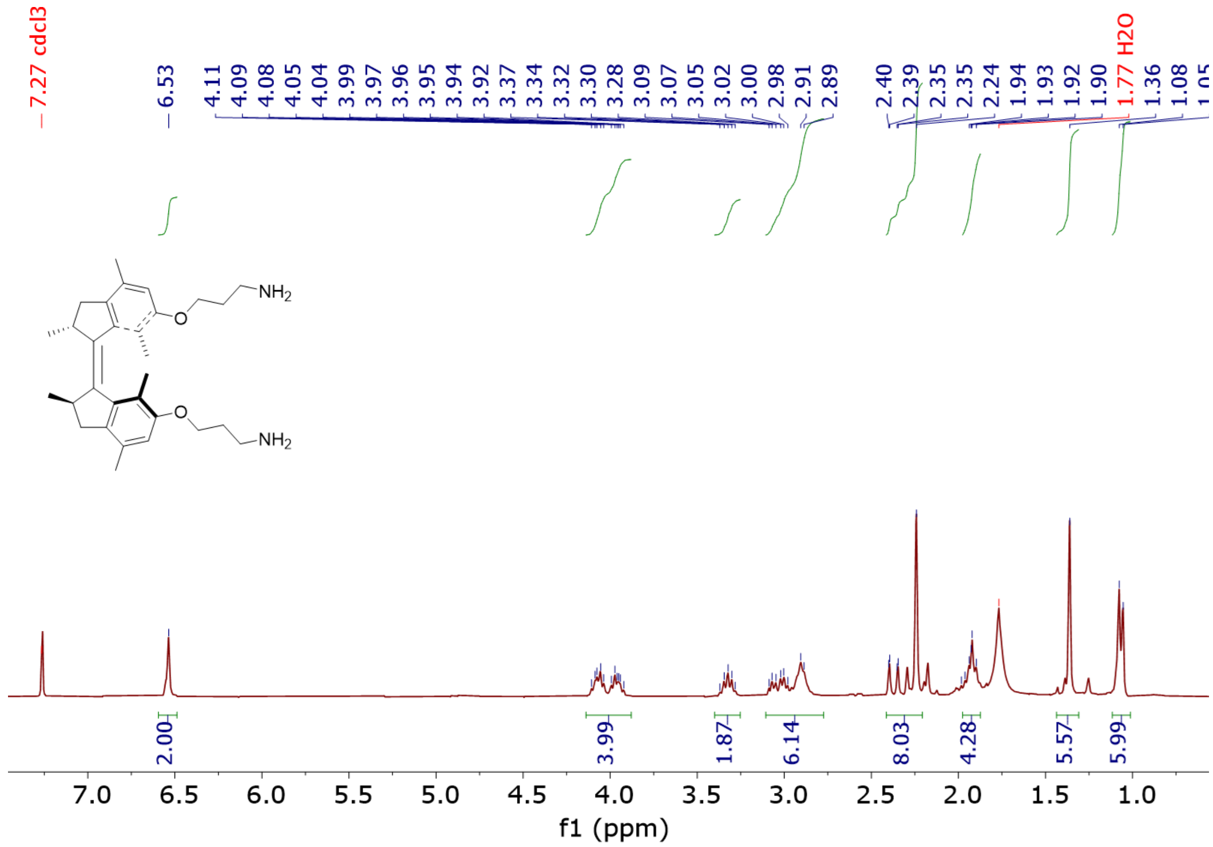


**Figure S27**. $^{1}$H NMR spectrum of (*P*,*P*)-*cis*-**3** ($CDCl_3$, 25 °C, 400 MHz).

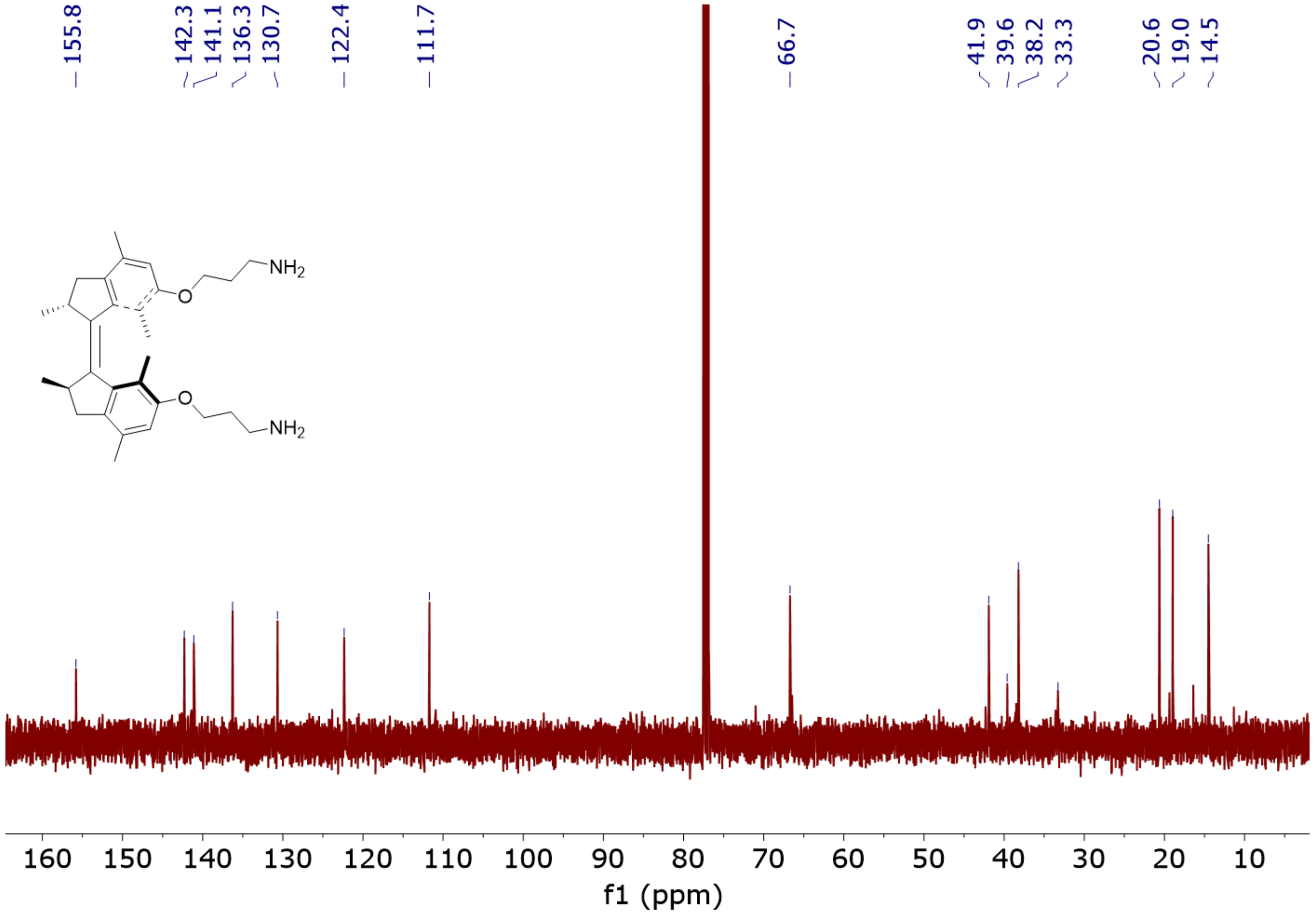


**Figure S28**. $^{13}$C NMR spectrum of (*P*,*P*)-*cis*-**3** ($CDCl_3$, 25 °C, 151 MHz).

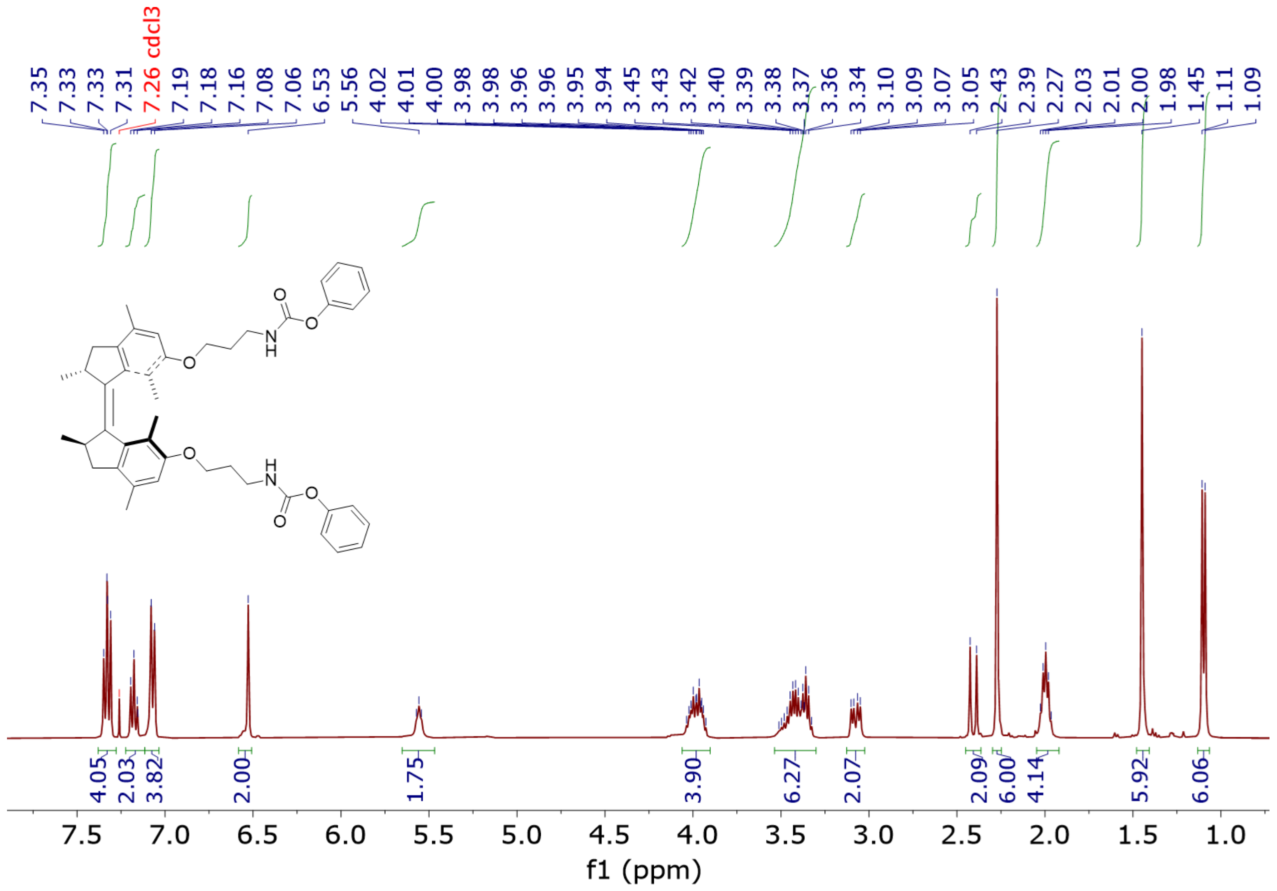

**Figure S29**. $^{1}$H NMR spectrum of (*P*,*P*)-*cis*-**4** ($CDCl_3$, 25 °C, 400 MHz).

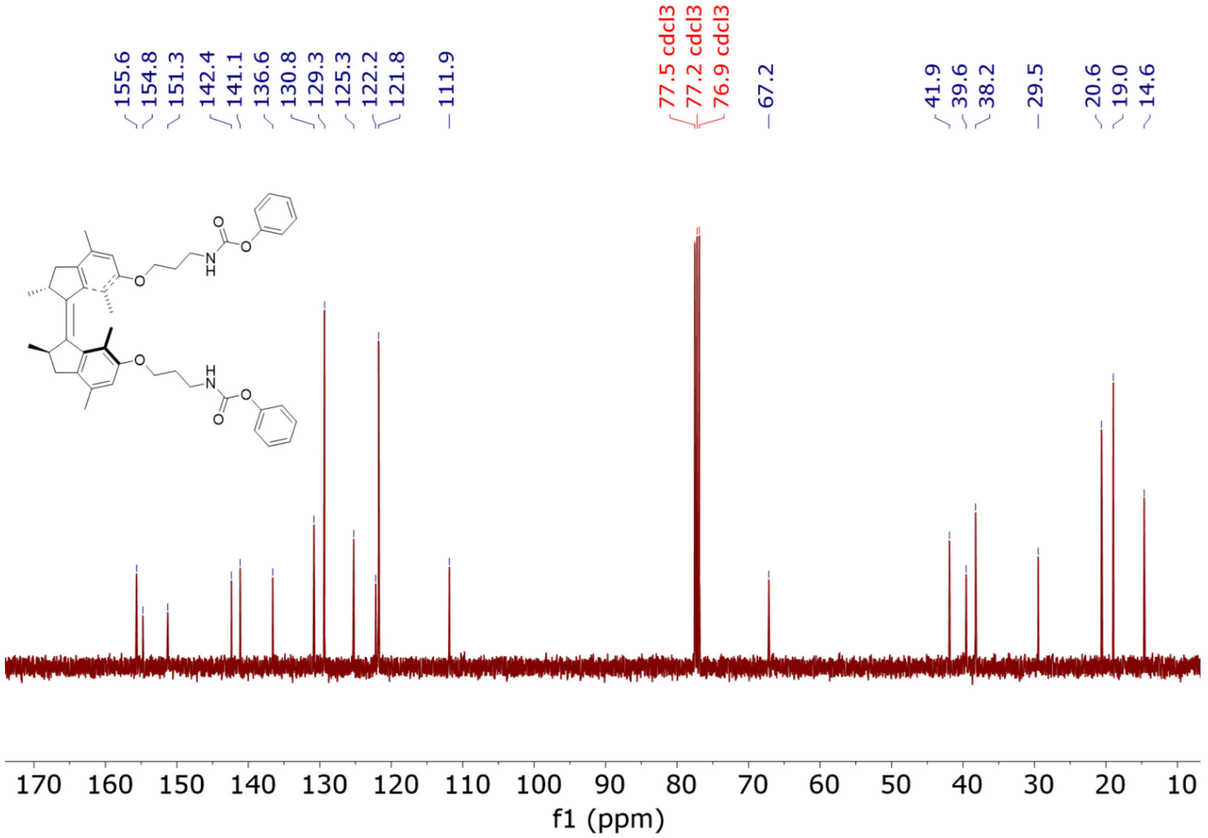


**Figure S30**. $^{13}$C NMR spectrum of (*P*,*P*)-*cis*-**4** ($CDCl_3$, 25 °C, 101 MHz).

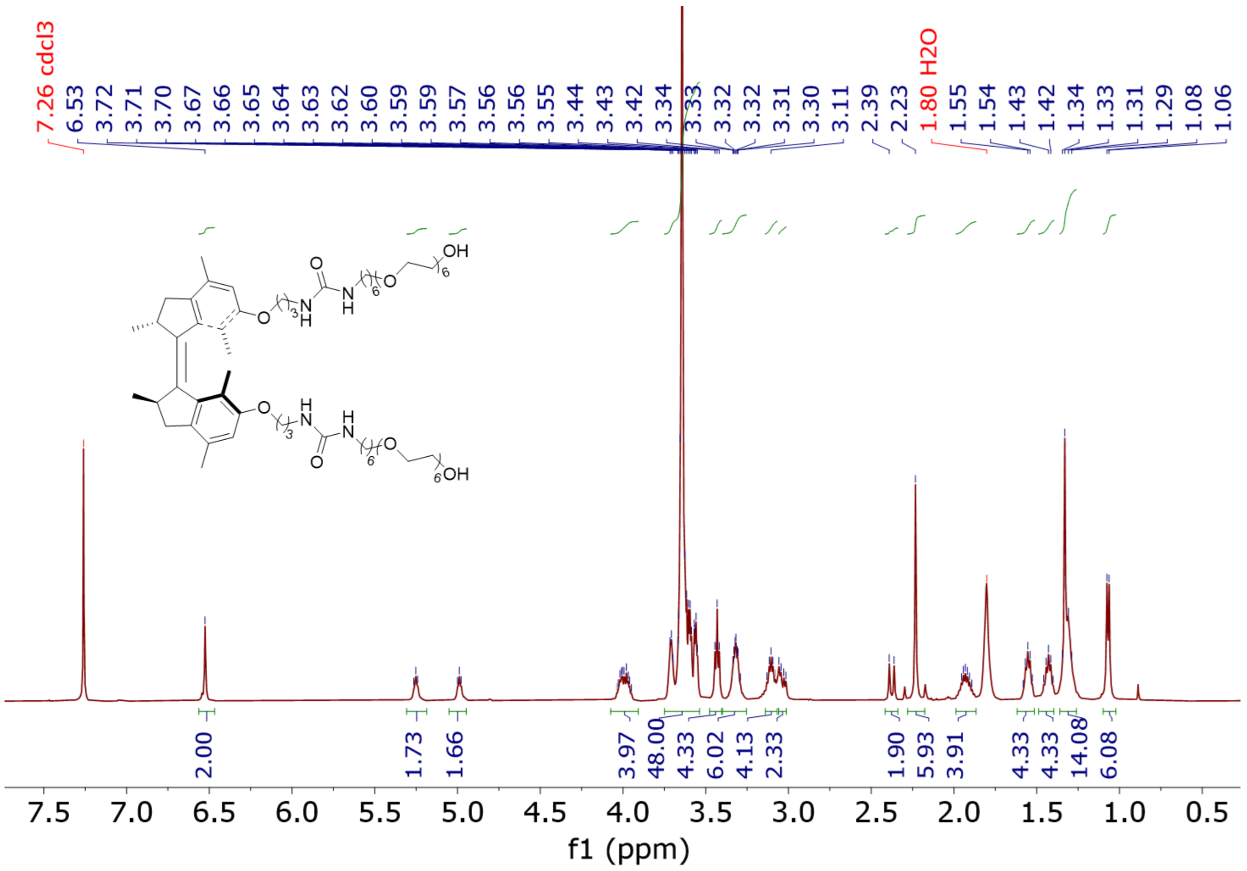


**Figure S31**. $^{1}$H NMR spectrum of (*P*,*P*)-*cis*-**M1**($CDCl_3$, 25 °C, 400 MHz).

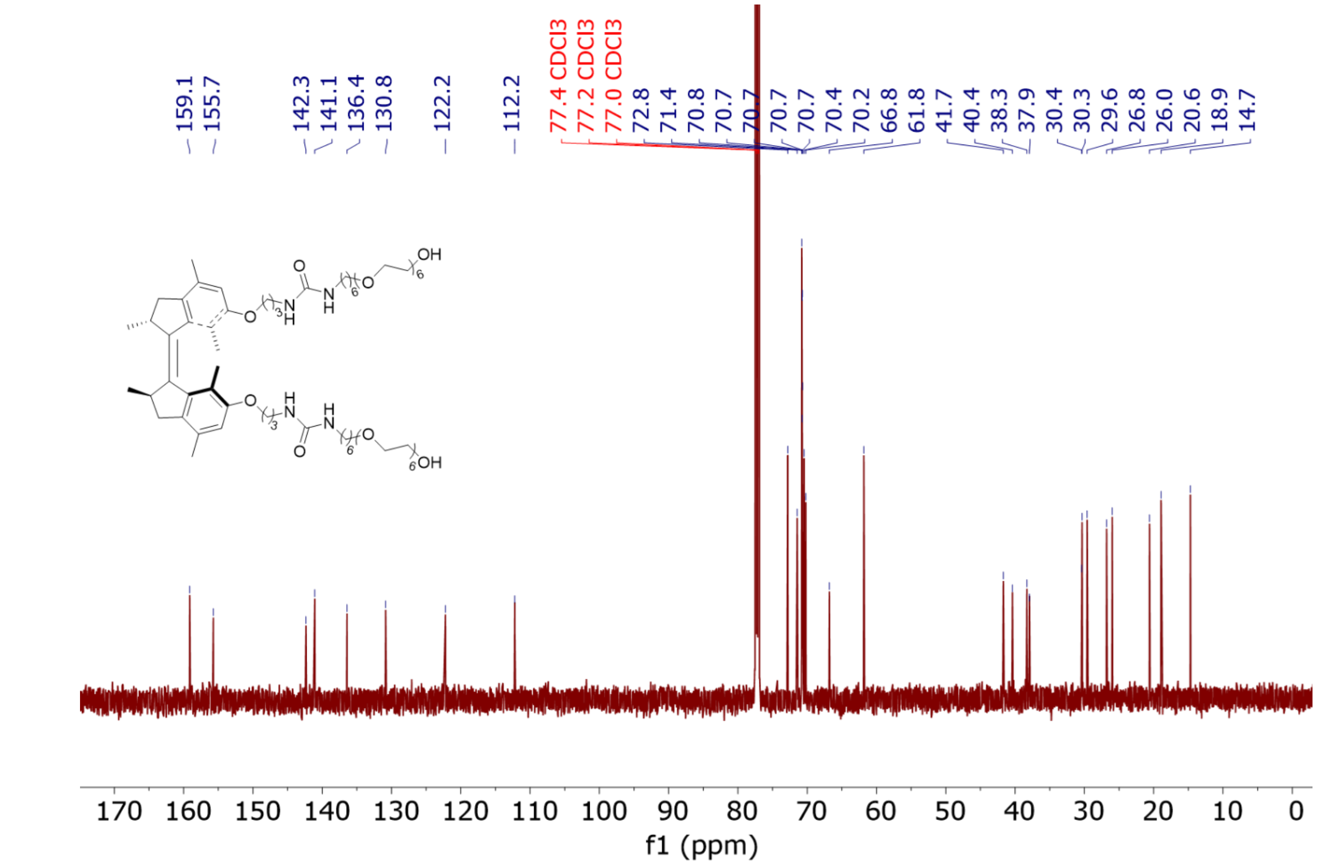


**Figure S32**. $^{13}C$ NMR spectrum of (*P*,*P*)-*cis*-**M1** ($CDCl_3$, 25 °C, 151 MHz).

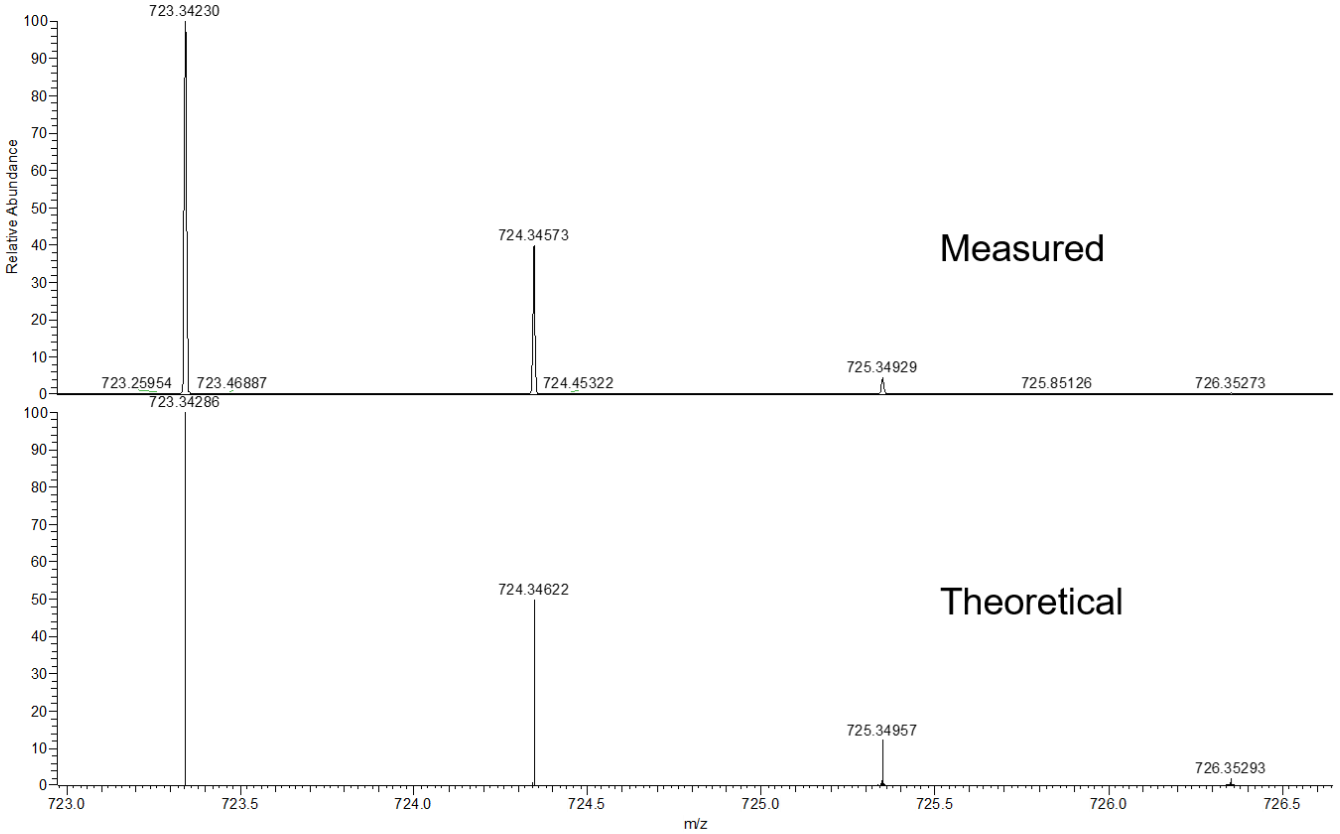


**Figure S33**. HRMS (ESI+) data of (*P*,*P*)-*cis*-**2** $[M+H]^+$, upper: measured data; lower: theoretical data.

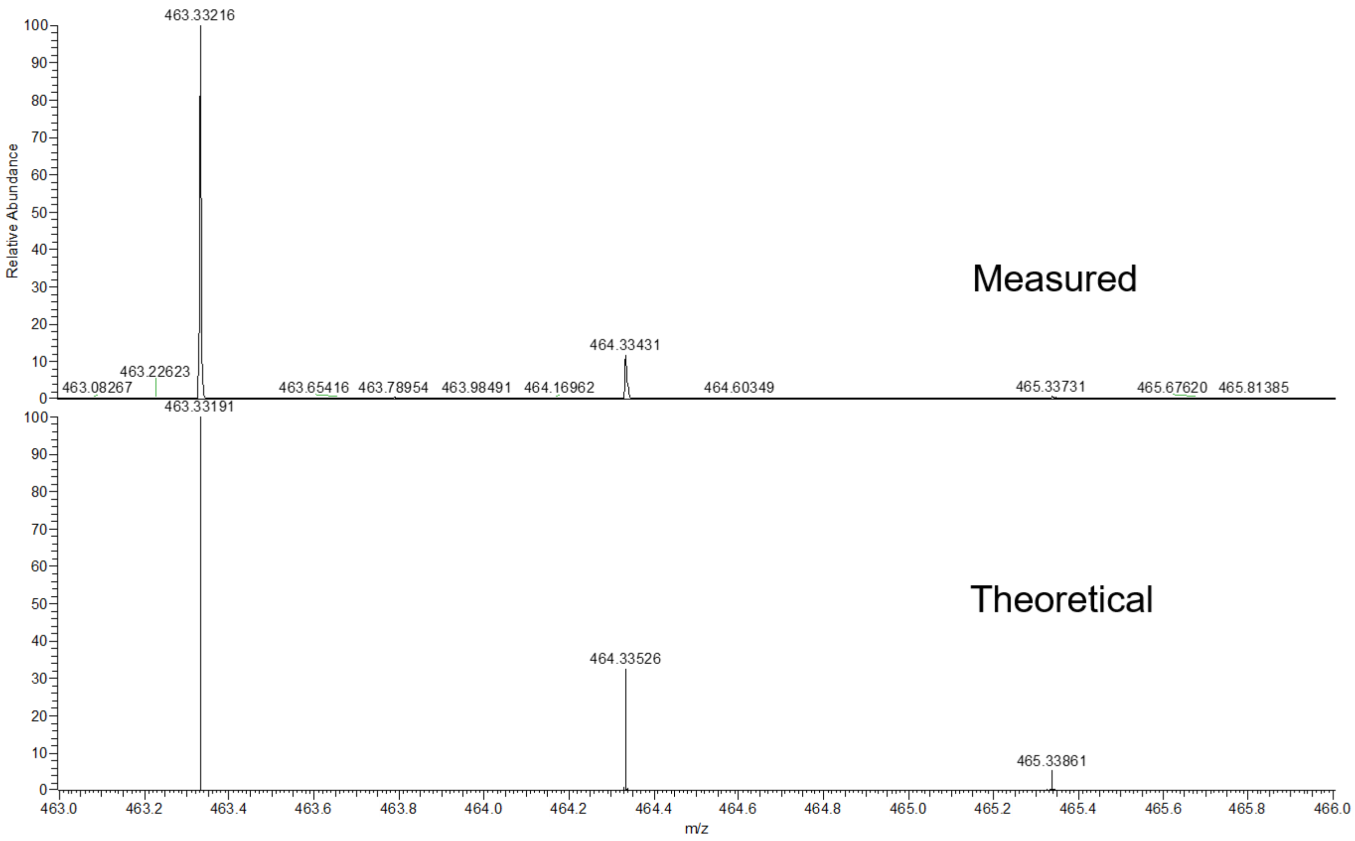


**Figure S34**. HRMS (ESI) data of (*P*,*P*)-*cis*-**3** [M+H]$^+$, upper: measured data; lower: theoretical data.

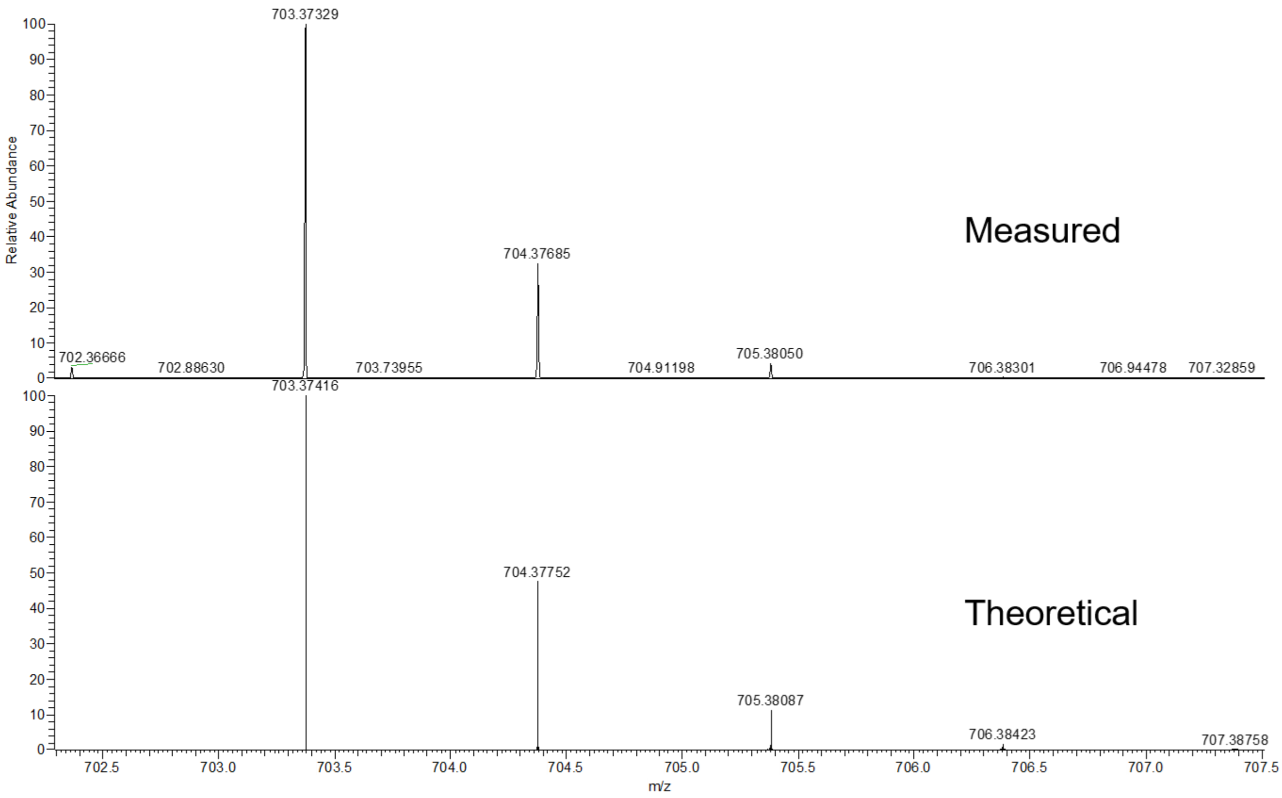


**Figure S35**. HRMS (ESI+) data of (*P*,*P*)-*cis*-**4** [M+H]$^+$, upper: measured data; lower: theoretical data.

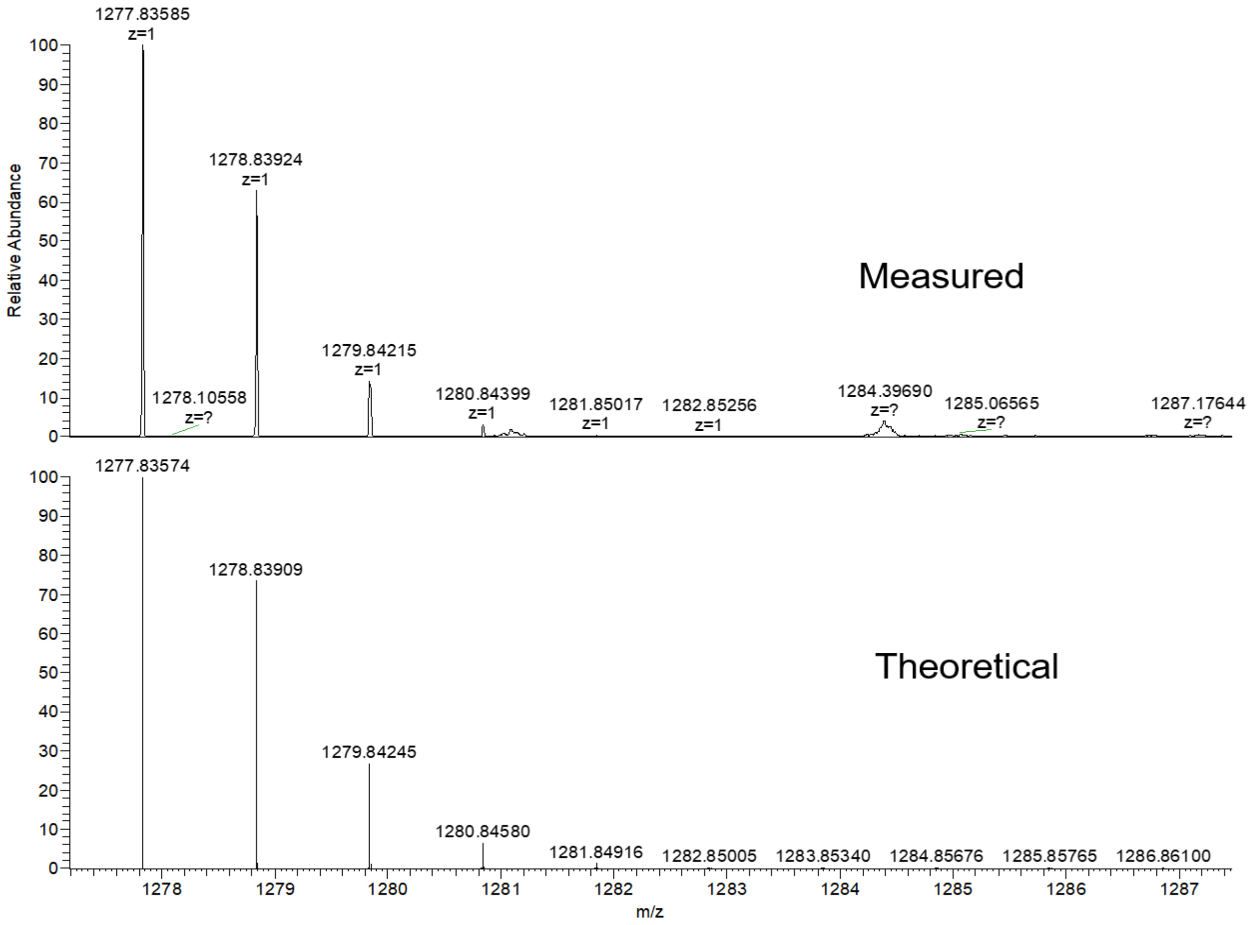


**Figure S36**. HRMS (ESI+) data of (*P*,*P*)-*cis*-**M1** $[M+H]^+$, upper: measured data; lower: theoretical data.

**References:**


(1) van Leeuwen, T.; Gan, J.; Kistemaker, J. C. M.; Pizzolato, S. F.; Chang, M.; Feringa, B. L. Enantiopure Functional Molecular Motors Obtained by a Switchable Chiral-Resolution Process. *Chem. A Eur. J.* **2016**, *22*, 7054–7058.

(2) Xu, F.; Pfeifer, L.; Crespi, S.; Leung, F. K. C.; Stuart, M. C. A.; Wezenberg, S. J.; Feringa, B. L. From Photoinduced Supramolecular Polymerization to Responsive Organogels. *J. Am. Chem. Soc.* **2021**, *143* (15), 5990–5997.

(3) Zhao, D.; van Leeuwen, T.; Cheng, J.; Feringa, B. L. Dynamic Control of Chirality and Self-Assembly of Double-Stranded Helicates with Light. *Nat. Chem.* **2016**, *9* (3), 250–256.

(4) Smulders, M. M. J.; Schenning, A. P. H. J.; Meijer, E. W. Insight into the Mechanisms of Cooperative Self-Assembly: The “Sergeants-and-Soldiers” Principle of Chiral and Achiral C3-Symmetrical Discotic Triamides. *J. Am. Chem. Soc.* **2008**, *130* (2), 606–611.

(5) Smulders, M. M. J.; Nieuwenhuizen, M. M. L.; de Greef, T. F. A.; van der Schoot, P.; Schenning, A. P. H. J.; Meijer, E. W. How to Distinguish Isodesmic from Cooperative Supramolecular Polymerisation. *Chem. A Eur. J.* **2010**, *16* (1), 362–367.

(6) Gillissen, M. A. J.; Koenigs, M. M. E.; Spiering, J. J. H.; Vekemans, J. A. J. M.; Palmans, A. R. A.; Voets, I. K.; Meijer, E. W. Triple Helix Formation in Amphiphilic Discotics: Demystifying Solvent Effects in Supramolecular Self-Assembly. *J. Am. Chem. Soc.* **2014**, *136* (1), 336–343.

(7) Wei, P.; Cook, T. R.; Yan, X.; Huang, F.; Stang, P. J. A Discrete Amphiphilic Organoplatinum(II) Metallacycle with Tunable Lower Critical Solution Temperature Behavior. *J. Am. Chem. Soc.* **2014**, *136* (44), 15497–15500.

(8) Mangold, C.; Obermeier, B.; Wurm, F.; Frey, H. From an Epoxide Monomer Toolkit to Functional

PEG Copolymers with Adjustable LCST Behavior. *Macromol. Rapid Commun.* **2011**, *32* (23), 1930–1934.
(9) Kholodenko, A. L. Analytical Calculation of the Scattering Function for Polymers of Arbitrary Flexibility Using the Dirac Propagator. *Macromolecules* **1993**, *26* (16), 4179–4183.
(10) Jaspers, M.; Pape, A. C. H.; Voets, I. K.; Rowan, A. E.; Portale, G.; Kouwer, P. H. J. Bundle Formation in Biomimetic Hydrogels. *Biomacromolecules* **2016**, *17* (8), 2642–2649.
(11) de Almeida, P.; Jaspers, M.; Vaessen, S.; Tagit, O.; Portale, G.; Rowan, A. E.; Kouwer, P. H. J. Cytoskeletal Stiffening in Synthetic Hydrogels. *Nat. Commun.* **2019**, *10* (1), 609.
(12) Guinier, A.; Fournet, G.; Yudowitch, L. K. *Small Angle Scattering of X-Rays*; Wiley: New York, 1955.
(13) Bressler, I.; Kohlbrecher, J.; Thunemann, A. F. SASfit: A Tool for Small-Angle Scattering Data Analysis Using a Library of Analytical Expressions. *J. Appl. Crystallogr.* **2015**, *48* (5), 1587–1598.
(14) Xu, F.; Pfeifer, L.; Stuart, M. C. A.; Leung, F. K. C.; Feringa, B. L. Multi-Modal Control over the Assembly of a Molecular Motor Bola-Amphiphile in Water. *Chem. Commun.* **2020**, *56* (54), 7451–7454.
(15) Vlatkovic, M.; Feringa, B. L.; Wezenberg, S. J. Dynamic Inversion of Stereoselective Phosphate Binding to a Bisurea Receptor Controlled by Light and Heat. *Angew. Chem. Int. Ed.* **2016**, *55* (3), 1001–1004.
(16) Wang, J.; Hou, L.; Browne, W. R.; Feringa, B. L. Photoswitchable Intramolecular Through-Space Magnetic Interaction. *J. Am. Chem. Soc.* **2011**, *133* (21), 8162–8164.
